# Systematic benchmarking of deep-learning methods for tertiary RNA structure prediction

**DOI:** 10.1371/journal.pcbi.1012715

**Published:** 2024-12-30

**Authors:** Akash Bahai, Chee Keong Kwoh, Yuguang Mu, Yinghui Li

**Affiliations:** 1 School of Biological Sciences (SBS), Nanyang Technological University, Singapore, Singapore; 2 School of Computer Science and Engineering, Nanyang Technological University, Singapore, Singapore; National University of Singapore, SINGAPORE

## Abstract

The 3D structure of RNA critically influences its functionality, and understanding this structure is vital for deciphering RNA biology. Experimental methods for determining RNA structures are labour-intensive, expensive, and time-consuming. Computational approaches have emerged as valuable tools, leveraging physics-based-principles and machine learning to predict RNA structures rapidly. Despite advancements, the accuracy of computational methods remains modest, especially when compared to protein structure prediction. Deep learning methods, while successful in protein structure prediction, have shown some promise for RNA structure prediction as well, but face unique challenges. This study systematically benchmarks state-of-the-art deep learning methods for RNA structure prediction across diverse datasets. Our aim is to identify factors influencing performance variation, such as RNA family diversity, sequence length, RNA type, multiple sequence alignment (MSA) quality, and deep learning model architecture. We show that generally ML-based methods perform much better than non-ML methods on most RNA targets, although the performance difference isn’t substantial when working with unseen novel or synthetic RNAs. The quality of the MSA and secondary structure prediction both play an important role and most methods aren’t able to predict non-Watson-Crick pairs in the RNAs. Overall among the automated 3D RNA structure prediction methods, DeepFoldRNA has the best prediction results followed by DRFold as the second best method. Finally, we also suggest possible mitigations to improve the quality of the prediction for future method development.

## Author summary

- Systematic benchmarking of five latest deep-learning and two fragment-assembly based methods on diverse datasets
- Compiled a new balanced dataset with latest RNA structures for benchmarking
- Generally, the ML-based methods outperform the traditional fragment-assembly based methods with DeepFoldRNA having the best predicted models overall
- On orphan RNA’s, the ML-based methods are only slightly better than FA-based methods, and generally all methods have poor performance on orphan RNAs.
- The performance of the methods is dependent on the MSA depth, RNA type, and secondary structure.

## Introduction

RNA molecules are essential players in various cellular processes, extending beyond their initial role as passive carriers of genetic information. Their diverse functions, including gene expression regulation, enzymatic activity, and regulatory mechanisms, have highlighted the importance of understanding RNA at a structural level [1]. The three-dimensional (3D) structure of RNA plays a critical role in determining its function [2]. Unlike DNA, RNA molecules can act as single-stranded entities, folding into intricate, dynamic architectures that dictate their biological activity and interactions with other molecules [3]. The 3D structure of RNA is vital for comprehending its diverse functions as the specific spatial arrangement of bases, loops, and stems within RNA molecules influences their ability to interact with proteins, small molecules, and other RNAs [4]. This structural organization enables RNA to perform critical tasks, such as serving as ribozymes (catalytic RNA molecules) or regulating gene expression by binding to target mRNA sequences [5,6]. In some cases, small changes in RNA’s 3D structure can significantly impact its function, making accurate structural prediction crucial for understanding RNA biology [7].

While there are experimental methods, such as X-ray crystallography, Nuclear Magnetic Resonance (NMR) spectroscopy, and cryo-electron microscopy (Cryo-EM) to determine RNA’s 3D structures, these methods are time-consuming, labour-intensive, and expensive [8,9]. As a result, they are often limited to studying only a fraction of the vast number of RNA molecules present in cells [10,11]. Moreover, RNA structures are inherently more challenging to resolve due to their dynamic nature, where they can adopt multiple conformations under different cellular conditions [12,13]. Given the challenges and limitations of experimental methods, computational approaches have emerged as essential tools in predicting RNAs 3D structure [14]. These computational methods leverage existing experimental data, principles of physics, and statistical analyses combined with machine learning to generate plausible models of RNA structures rapidly and cost-effectively [15]. By simulating the folding process, these techniques can provide valuable insights into the energetics and conformational landscape of RNA molecules [16].

Various computational methods have been developed for RNA structure prediction [17]. They can be either *ab initio*, which try to predict the structure from scratch or template-based, which predict the structure based on information from already known structures. The *ab initio* approaches generally rely on coarse-grained approach to simulate the folding using molecular dynamics and search for the conformation with the lowest free energy [18–26]. The template-based methods include fragment-based-assembly (FA-based), comparative modeling, homology modeling, and threading-based approaches, which exploit the sequence and structural similarities between known RNA structures and the target RNA, to predict its 3D structure [27–35]. Some methods combine the coarse-grained folding and fragment-based (FA) modeling approaches [36–39]. However, despite significant progress, the accuracy of computational methods for RNA structure prediction remains relatively modest when compared to the progress in computational protein structure prediction [40–42]. The problem of RNA 3D structure prediction shares many similarities with protein structure prediction, which has been extensively studied [43]. However, predicting RNA structures is often considered more challenging due to several factors. Unlike proteins, there is a relative paucity of experimentally determined RNA structures in publicly available databases (there are only approximately 1700 RNA-only non-homologous structures in the Protein Data Bank), and structures of RNAs from majority of the RNA families haven’t been experimentally determined (only 87 of the 2791 RNA families have one or more solved 3D structure) [44,45]. This lack of available data makes it harder to establish reliable templates for comparative modelling. Additionally, RNA structures are more dynamic, and their folding pathways are influenced by a myriad of factors with complicated secondary structures, further complicating the prediction process [46]. In recent years, deep-learning-based methods have shown promise in various bioinformatics tasks, including protein structure prediction [47]. AlphaFold has revolutionized the field of computational structural modelling, following which, researchers have started exploring the application of similar deep-learning (DL) methods to tackle the challenges of 3D RNA structure prediction [48–50]. The emergence of the end-to-end sequence-based DL methods have shown promise by directly predicting RNA structures from sequence data. They have the advantage of bypassing the need for explicit template structures or extensive feature engineering. DL models, particularly those based on transformer networks and attention mechanisms, can capture long-range complex dependencies within RNA sequences, which are crucial for accurate structure prediction [51–55].

However, it is worth noting that in the last conducted CASP 15 (Critical Assessment of Techniques for Protein Structure Prediction), AI-based methods did not perform as well in RNA structure prediction task compared to their performance in protein structure prediction tasks [56,57]. This highlights the unique challenges posed by RNA structures. Several factors, such as the diversity of RNA families, the varying lengths of RNA molecules, the specific type of RNA (e.g., tRNA, rRNA, mRNA), the degree of sequence homology, and the quality of multiple sequence alignments (MSA), could contribute to the variance in performance. In this study, we systematically benchmark the latest deep-learning-based methods for RNA structure prediction across multiple datasets. Our goal is to investigate the factors contributing to the performance variance, including RNA family diversity, sequence length, RNA type, and MSA quality. We are only focusing on benchmarking the methods out-of-the-box based on their automated prediction capabilities, with no human intervention or human knowledge input.

At the conclusion of our study, we provide valuable insights into how DL-based methods for RNA structure prediction can be improved. The results from our independent benchmarking offer guidance on the selection of the most suitable method for different RNA structure prediction use cases. To provide a comprehensive perspective, we have also included two baseline comparisons to non-DL methods, offering a holistic view of the current landscape of 3D RNA structure prediction. Through this systematic benchmarking, we aim to enhance our understanding of the capabilities and limitations of AI-driven approaches in deciphering the 3D structures of RNA molecules, ultimately contributing to advancements in RNA biology and related fields.

## Methods

### RNA Structure prediction methods

We compiled a list of DL-based methods for RNA structure prediction in the recent literature and implemented them locally on our systems to allow a large-scale comparison for multiple targets (Table 1). We also included two fragment-assembly based methods (non-ML) to allow an overall comparison of deep-learning methods against the traditional methods. The details of the benchmarked methods are provided in the Table 1. In the following, we describe the methods in more details.

**Table 1:** The five deep-learning (DL-based) methods and two fragment-assembly (FA-based) methods included in the benchmarking. The MSA columns indicates whether the DL-based methods include MSA as input and the Secondary structure column indicates whether the method uses secondary structure as input. The methods used to predict the MSA and secondary structure are provided in the parenthesis in each column respectively.

Method	Type	MSA	Secondary structure	Year
**RosettaFold2NA**	Deep learning (ML)	Yes (rMSA lite)	No	2022
**DeepFoldRNA**	Deep learning (ML)	Yes (rMSA)	Yes (PETfold)	2022
**DRFold**	Deep learning (ML)	No	Yes (PETfold + RNAfold)	2022
**RhoFold**	Deep learning (ML)	Yes (blastn)	No	2022
**trRosettaRNA**	Deep learning (ML)	Yes (rMSA + Infernal)	Yes (SPOT-RNA)	2022
**RNAComposer**	Fragment-assembly (non-ML)	-	Yes (RNAfold)	2012
**3DRNA**	Fragment-assembly (non-ML)	-	Yes (RNAfold)	2012

### RosettaFold2NA

RoseTTAFold2NA [58] is a deep learning-based method for predicting the 3D structure of RNA and protein-nucleic acid complexes. It is an extension of the original RoseTTAFold [59,60] method, which was developed for protein structure prediction. RoseTTAFold2NA uses a single trained network to produce 3D structure models with confidence estimates for protein-DNA and protein-RNA complexes, and for RNA tertiary structures. The architecture of RoseTTAFold2NA is based on a deep neural network that combines convolutional and recurrent layers to capture both local and global features of the input sequences. The input to the network consists of multiple sequence alignments (MSAs) of related protein and nucleic acid molecules, which are generated using sequence similarity searches against RNA sequence databases using rMSA lite [61].

### DeepFoldRNA

DeepFoldRNA is a fully automated method for predicting RNA tertiary structures from sequence alone using deep self-attention neural networks to predict geometric restraints. It takes the MSA and predicted secondary structure as input and then uses a self-attention-based neural network architecture to predict geometric restraints, which are then used to guide the construction of 3D RNA structures through limited-memory Broyden-Fletcher-Goldfarb-Shanno (L-BFGS) minimization simulations. The geometric potential is derived from the predicted distances between pairs of atoms in the RNA molecule. Specifically, the predicted geometric restraints include pairwise distance maps between the N1/N9 atoms, C4’ atoms, and backbone P atoms, as well as inter-residue and backbone torsion angles (*ω*, *λ*, *η*, *θ*). These predicted geometric restraints are converted into composite potentials by taking the negative log-likelihood of the binned probability predictions, which are then used to guide the L-BFGS folding simulations.

### DRFold

DRfold is a novel deep learning-based method for *ab initio* RNA structure prediction that uses self-attention transformer networks to learn coarse-grained RNA structures directly from sequence. DRfold adopts a coarse-grained model of RNA specified by the phosphate P, ribose C4’, and glycosidic N atom of the nucleobase for training efficiency. The predicted conformations are further optimized by a separately trained deep-geometric potential through gradient-descent based simulations.

### RhoFold

RhoFold is an end-to-end deep learning method for accurate de novo RNA 3D structure prediction. It utilizes multi-aspect information of the RNA sequence to infer the 3D structure, including multiple sequence alignment (MSA) and information from a newly proposed RNA foundation model (RNA-FM). The method also introduces secondary structure information into the loss function and employs a novel procedure to perform self-distillation. The pipeline consists of three main components: the feature extraction module, the structure prediction module, and the structure refinement module. The entire model is fully differentiable, and several additional constraints are added to the structure output to ensure the final output is valid without clashing structures. The feature extraction module uses an RNA foundation model (RNA-FM) and a 4-layer E2Eformer module to learn the sequence representations and interactions between different nucleotides. The structure prediction module uses an 8-layer structure module to generate the final RNA 3D structures. Finally, the structure refinement module employs a recycling technique similar to AlphaFold to enhance prediction accuracy.

### trRosettaRNA

This method is built on top of trRosetta method (for protein structure prediction) [62] and is adapted for RNAs. The authors used rMSA to build a MSA and SPOT-RNA method for secondary structure prediction. The deep-learning architecture of the method is a transformer network (named RNA-former) that takes MSA and pair representation as input and predicts 1D and 2D geometries, which are then converted into restraints to guide the 3D structure folding step. The full-atom structure of the RNA is generated by energy minimization with deep learning potentials and physics-based energy terms from Rosetta. The authors also constructed a self-distillation dataset by collecting the bpRNA sequences from the Rfam database.

### RNAComposer

This is a fragment-assembly based method that takes the RNA sequence and secondary structure as input to predict the RNA 3D structure. It works by breaking the secondary structure of the RNA into multiple substructures, including helices, loops, and bulges. It then searches for matches for each of the substructures in a database of known helices and loops from experimentally determined 3D structures, called RNA FRABASE [63]. The matches are selected based on the secondary structure topology, sequence similarity and source structure resolution. The 3D structure is then assembled by superimposing common Watson-crick pairs at the ends of the substructures to combine all the substructures together and get the full-length 3D structure. The assembled structure is refined and energy minimized to obtain the final predicted model. The webserver for this method is available at http://rnacomposer.ibch.poznan.pl/. The webserver provides multiple choices for the method to be used to predict the secondary structure. However, we used RNAFold [64] method to predict the input secondary structure for our benchmarking.

### 3DRNA

This method also works similar to RNAComposer i.e. using secondary structure elements as building blocks to assemble the complete 3D structure of the RNA. The secondary structure is broken down into substructures of helices, different loops, and pseudoknots. These substructures are searched in a library of 3D structure-based templates assembled from known experimental RNA structures. In case of no matches, the templates are built using Bi-residue or distance geometry algorithm. The matched templates are then assembled to create initial 3D structures, which are put through a simulated annealing Monte Carlo process. This involves iteratively translating or rotating randomly selected movable elements, incorporating experimental restraints when available, and clustering the resulting structures using k-means clustering. The centroids of the clusters can be scored using a statistical potential to rank the structures and select the best predictions. The method is available at http://biophy.hust.edu.cn/new/3dRNA. The method can use multiple methods to predict the secondary structure, and we used RNAFold again to predict the secondary structure in this benchmarking.

### Datasets

#### CASP15 RNA

In the 2022 edition of CASP, RNA structure prediction was introduced as a prediction category for the first time [65]. RNA-Puzzles experiment has been a long-running CASP-style experiment for RNAs from 2010 to 2021 during which the organizers evaluated 22 RNA-Puzzles challenge [66–69]. In 2022, RNA-Puzzles joined forces with CASP to expand the target and predictors base and stimulate interest in RNA structure prediction field within the protein prediction community. The integration of RNA structure prediction into CASP reflects the growing recognition of the importance of RNA in cellular processes and the need for effective computational tools to predict RNA structures reliably. There were 12 targets selected this year which covered natural RNAs, synthetic RNAs, and RNA-protein complexes (Table 2). We selected this dataset as none of these RNAs are part of the training dataset of the DL-methods being benchmarked. Generally, it’s much more difficult to predict the structure of RNA-protein complexes and synthetic RNAs because most of the prediction methods are only trained on single chain RNAs and synthetic RNAs don’t have homologous sequences in the RFam database [70] so the MSA isn’t very informative.

**Table 2:** The targets included in the CASP15 dataset. There are 13 targets in total, out of which four are synthetic and rest are natural RNAs. The difficulty of the targets is taken as estimated by the CASP15 organizers. Four of the targets are X-ray crystallography structures while the remaining nine are Cryo-EM structures. R1189 and R1190 are protein-RNA complex structures which are generally much harder to predict compared to unbound RNAs.

Name	Type	Length	Experimental Method (resol Å)	RNA Type	Difficulty
R1107	RNA	69	X^a^ (2.8)	CPEB3 ribozyme	Natural/medium
R1108	RNA	69	X (2.2)	CPEB3 ribozyme	Natural/medium
R1116	RNA	157	X (3.0)	Cloverleaf RNA	Natural/difficult
R1117	RNA-ligand	30	X (2.3)	PreQ1 class I type III riboswitch	Natural/easy
R1126	RNA-ligand	363	E^b^ (5.6)	Traptamer	Synthetic
R1128	RNA	238	E (5.3)	Paranemic Crossover Triangle	Synthetic
R1136	RNA-ligand	374	E (4.5)	Apta-FRET	Synthetic
R1138	RNA	720	E (5.2)	6HBC	Synthetic
R1149	RNA	124	E (4.7)	SARS-CoV-2 SL5	Natural/difficult
R1156	RNA	135	E (5.8)	BtCoV-HKU5 SL5	Natural/difficult
R1189	RNA-protein	173	E (3.8)	RsmZ-RsmA	Natural/difficult
R1190	RNA-protein	173	E (4.6)	RsmZ-RsmA	Natural/difficult

#### New dataset

We compiled a new dataset of recently published RNA structures in the PDB database [71] to create a more rigorous test of the prediction capabilities of the selected methods (Table 3). We only included RNAs that have been published after the publication of these tools (late 2022) to ensure that none of the RNAs have been included in the training dataset of these methods. This dataset includes RNAs of different types including structures from single RNAs, RNA-ligand complexes, RNA-protein complexes, and synthetic RNAs thus covering the diversity of the RNA structure prediction field. As the methods we benchmarked can only make prediction on single chain RNAs (except RosettaFold2NA), we only made structure predictions on single RNA sequences while discarding the other chains. Our final dataset had 24 targets out of which 16 were natural RNAs and 8 were synthetic. The length of the target RNAs varies from very small RNAs (only 14 nucleotides long) to much longer RNAs (426 nucleotides long). Most of these recently published RNAs have been determined using Cryo-electron microscopy (14/24) [72], with the remaining ones using X-Ray crystallography (9/24) [73] and one using solution NMR [74]. This set of RNAs should serve as one of the most updated and stringent benchmarking dataset for existing and newly developed RNA structure prediction methods.

**Table 3:** Targets compiled in the new RNA dataset. ^a^X = X-Ray crystallography, ^b^E = Cryo-electron microscopy. All the targets were selected based on their deposition date in the PDB database to make sure that none of the benchmarking-methods contain these targets in their training set.This dataset is well-balanced with 14/24 coming from Cryo-EM and 9/24 coming from X-ray crystallography. 16 of the targets are natural RNAs while 8 are synthetic RNAs.

Name	Type	Length	Experimental Method (resol Å)	RNA Type	Difficulty
7qr4	RNA-protein	69	X^a^ (2.83)	CPEB3 HDV-like ribozyme	Natural
7qsh	RNA-ligand	25	X (0.86)	23S ribosomal RNA	Natural
7u0y	RNA-protein	67	X (2.66)	Pepper RNA aptamer	Natural
7ur5	RNA	90	E^b^ (2.60)	allo-tRNAUTu1 of the E. coli ribosome	Synthetic
7uri	RNA	90	E (2.60)	allo-tRNAUTu1A of the E.coli ribosome	Synthetic
7wi9	RNA-ligand	50	X (2.98)	THF-II riboswitch	Natural
7wju	RNA-protein-DNA	191	E (2.69)	AsCas12f1-sgRNAv1-dsDNA ternary complex	Synthetic
7xd3	RNA-ligand	426	E (4.05)	Tetrahymena group I intron	Synthetic
7xjz	RNA-protein	74	E (3.90)	tRNA	Natural
7yoj	RNA-protein-DNA	174	E (3.36)	CasPi with guide RNA and target DNA	Natural
7yr6	RNA-protein	118	E (4.80)	RsmZ RNA	Natural
7zj5	RNA	374	E (4.55)	Aptamer FRET	Synthetic
8bf8	RNA-protein	150	E (2.80)	reRNA	Natural
8bgu	RNA-protein	121	E (4.10)	5S rRNA	Natural
8d9l	RNA-protein	73	E (4.04)	Lys-tRNA	Natural
8dp3	RNA-protein	90	X (1.91)	B3 cloverleaf RNA	Synthetic
8ex9	RNA-protein-DNA	150	E (2.96)	reRNA	Natural
8eyu	RNA-ligand	49	X (1.95)	aptamer Beetroot	Synthetic
8f4o	RNA-ligand	83	X (3.10)	riboswitch aptamer	Natural
8fcs	RNA	71	NMR	microRNA	Natural
8fzr	RNA-protein	77	E (3.60)	Arg tRNA	Natural
8gxb	RNA-protein	61	X (2.15)	NAD+ -II riboswitch	Natural
8h1j	RNA-protein-DNA	247	E (3.10)	omegaRNA	Natural
8sxl	RNA-ligand	14	X (1.90)	RNA UU template	Synthetic

### RNA-Puzzles dataset

The RNA-Puzzles dataset (Table 4) is a collection of experimentally determined 3D RNA structures that are used in the critical assessment of 3D RNA structure prediction methods, known as the "RNA-Puzzles" experiment [75]. RNA-Puzzles is an ongoing collaborative experiment and community effort in the field of structural biology which aims to assess the state of the art in RNA tertiary structure prediction. It is inspired from the CASP experiment for protein structure prediction. The dataset consists of RNA structures whose 3D coordinates have been determined through experimental techniques such as X-ray crystallography, NMR spectroscopy, or cryo-electron microscopy. These experimental structures serve as ground truth for assessing the quality of predicted structures. RNA-Puzzles includes RNA structures from various categories, including natural RNA, synthetic RNA, and RNA-protein complexes, where each category presents unique challenges for structure prediction. Since most of the rounds of RNA-Puzzles were held before 2020 (up to puzzle 24), many of the targets in this dataset might have been part of the training set of the deep-learning methods that we are trying to benchmark, therefore, the benchmarking performance on this dataset could be overestimated. Also, out of the 36 puzzles in our dataset, 35 have been determined using X-ray crystallography, which means that the comparisons with the native structure are more informative about the capabilities of the prediction methods (X-ray crystal structures are more reliable than Cryo-EM or NMR structures). The latest RNA-Puzzles targets (published after 2022) are PZ34, PZ37 and PZ38 (PZ39 has already been included in our newly compiled dataset) and these targets should be comparatively more challenging for the prediction methods.

**Table 4:** Targets from the RNA-puzzles dataset. ^a^X = X-Ray crystallography, ^b^E = Cryo-electron microscopy. Most of these targets were published before the publications of the ML-base methods so it’s highly possible that these are part of the training data of those tools. This will make this dataset the easiest dataset for prediction and the reported performance of most methods might be overinflated on this dataset. Also, 35 out of the 36 targets are X-ray crystallography structures which are known to be more accurate and have better resolution, so the comparison of the predicted model with these native crystallographic structures is more accurate. The puzzle pz34, pz37 and pz38 have been published recently so these targets should be comparatively more challenging for our benchmarking methods.

Name	PDB Id	Type	Length	Experimental Method (resol Å)	RNA Type	Difficulty
**pz1**	3MEI	RNA-ligand	23	X^a^ (1.97)	thymidylate synthase mRNA	Natural
**pz2**	3P59	RNA-ligand	15	X (2.18	RNA Nanosquare	Natural
**pz3**	3OWZ	RNA-ligand	84	X (2.95)	Glycine riboswitch	Natural
**pz4**	3V7E	RNA-Protein	126	X (2.80)	SAM-I riboswitch aptamer	Natural
**pz5**	4P9R	RNA-ligand	188	X (2.70)	Group I intron	Natural
**pz6**	4GXY	RNA-ligand	158	X (3.05)	Adenosylcobalamin riboswitch	Natural
**pz7**	4R4V	RNA-ligand	185	X (3.07)	Varkud satellite ribozyme	Natural
**pz8**	4L81	RNA-ligand	96	X (2.95)	SAM riboswitch	Natural
**pz9**	5KPY	RNA-ligand	71	X (2.00)	5-hydroxytryptophan aptamer	Synthetic
**pz10**	4LCK	RNA-Protein	96	X (3.20)	T-box riboswitch—tRNA complex	Natural
**pz11**	5LYS	RNA-ligand	57	X (2.32)	7SK 5’-hairpin—Gold derivative	Natural
**pz12**	4QLM	RNA-ligand	117	X (2.72)	ydaO riboswitch	Natural
**pz13**	4XW7	RNA-ligand	60	X (2.50)	ZMP Riboswitch	Natural
**pz14**	5DDO	RNA-Protein	58	X (3.10)	L-glutamine riboswitch	Natural
**pz15**	5DI4	RNA-ligand	48	X (2.95)	Hammerhead Ribozyme	Synthetic
**pz16**	6Y0Y	RNA-ligand	27	X (0.95)	Sarcin Ricin Loop	Synthetic
**pz17**	5K7C	RNA-Protein	58	X (2.73)	Pistol ribozyme	Synthetic
**pz18**	5TPY	RNA-ligand	71	X (2.81)	resistant RNA from Zika virus	Natural
**pz19**	5T5A	RNA-DNA	40	X (2.00)	One Twister Sister robozyme	Natural
**pz20**	5Y85	RNA-DNA	71	X (2.00)	4-way junctional Twister-Sister Ribozyme	Natural
**pz21**	5NWQ	RNA-ligand	41	X (1.91)	Guanidine III Riboswitch	Natural
**pz22**	6JQ5	RNA	82	X (2.06)	Hatchet Ribozyme	Synthetic
**pz23**	6E8U	RNA-ligand	37	X (1.55)	Mango-III aptamer	Synthetic
**pz24**	6OL3	RNA-ligand	112	X (2.74)	Adenovirus-associated RNA	Natural
**pz25**	6P2H	RNA-ligand	69	X (2.80)	2’-dG-II riboswitch	Synthetic
**pz26**	6PMO	RNA-ligand	66	X (2.66)	T-box riboswitch—tRNA complex	Natural
**pz27**	6POM	RNA	170	E^b^ (4.90)	T-box riboswitch—tRNA complex	Natural
**pz28**	6UFM	RNA-Protein	98	X (2.82)	T-box riboswitch—tRNA complex	Natural
**pz29**	6TB7	RNA-ligand	52	X (2.53)	NAD+ riboswitch	Natural
**pz30**	7BG9	RNA-Protein	88	E (3.80)	Human telomerase RNA	Synthetic
**pz31**	7MLX	RNA-Protein	65	X (2.09)	PFSE in Sars-CoV-2	Natural
**pz32**	7EOJ	RNA-ligand	49	X (1.77)	Pepper Aptamer	Synthetic
**pz33**	7ELP	RNA-ligand	46	X (2.79)	Xanthine riboswitch	Natural
**pz34**	7V9E	RNA-ligand	68	X (2.30)	Methyl transferase ribozyme	Natural
**pz37**	8GXC	RNA-Protein	61	X (2.50)	NAD+ II riboswitch	Natural
**pz38**	8HB8	RNA-Protein	55	X (2.30)	NAD+ II riboswitch	Natural

### RNA3DB Dataset

We compiled a new dataset to further evaluate the performance of the selected RNA structure prediction methods. This dataset comprises a non-redundant collection of orphan RNAs, which are structurally and sequentially independent RNA structures sourced from the RNA3DB database [76]. The RNA3DB database is a curated collection of structured RNAs from the Protein Data Bank (PDB), organized into non-redundant components to facilitate training and benchmarking of deep learning models in RNA structure prediction. We selected 20 targets from this dataset, focusing on small RNAs ranging from 22 to 59 nucleotides in length that were published after mid-2022. We have enforced a maximum sequence identity cutoff of 80% against structures in the PDB published before 2022 to select these targets using cd-hit [77]. The targets include a diverse array of RNA types, such as synthetic RNAs, small messenger RNAs crystallized as part of larger complexes, and viral RNAs. This selection of orphan RNAs should serves as a challenging benchmark for the prediction methods, as these structures do not belong to any known Rfam families [78] and lack homologous sequences in existing databases.

### Assessment metrics

We used root-mean-square deviation (RMSD) of atomic positions as the metric for quantifying similarity between the modelled and the native structure. RMSD is a distance metric which measures the average distance between the corresponding atoms of the equivalent residues of two superimposed structures [79]. The predicted 3D models might have different number of residues/atoms than the native structure, therefore we used the RNA-puzzle toolkit [80] to normalize the predicted structures to the native structures. Sometimes the native PDB file might have some part of the structure or residues missing, so we only compared the common residues in the model and native structures. Additionally, hydrogen atoms were removed from the structures to ensure consistency/standardization across the different predicted models and focus primarily on the backbone atoms. The RMSD between two structures v and w, with n atoms is defined as:

$$RMSD(v,w)=\sqrt{\frac{1}{n}\sum_{i=1}^{n}{\left(v_{ix}-w_{ix}\right)}^{2}+{\left(v_{iy}-w_{iy}\right)}^{2}+{\left(v_{iz}-w_{iz}\right)}^{2}}$$

Here, *ix*, *iy*, *iz* denote the cartesian coordinates of the *i*^*th*^ atom. We calculated the all-atom RMSD for comparing the structures, i.e., using all the atoms in the residues (except Hydrogens) instead of just heavy atoms or the backbone atoms. While root mean square deviation (RMSD) measurements are commonly employed to assess structural similarity, they may not provide a comprehensive evaluation of the accuracy of predicted 3D RNA structures in comparison to the native structure. For example, two RNA structures may exhibit similar all-atom RMSD values but differ in their base orientations, making RMSD alone an ambiguous criterion. To address this limitation, various metrics and assessment tools have been introduced [81]. These additional measures aim to provide a more nuanced and informative assessment of RNA structural predictions. Interaction network fidelity (INF) is a similarity metric that compares the Watson-Crick (WC) and non-Watson-Crick (nWC) base pairs in the models to the native structure and also the base-stacking interactions [82].


$$INF=\sqrt{PPV\times STY},\;where$$

$$PPV=\frac{\left|TP\right|}{\left|TP|+|FP\right|}\;and\;STY=\frac{\left|TP\right|}{\left|TP|+|FN\right|}$$

Here TP, FP, and FN are defined based on the absence of base-pairs in the native and modelled structures. We used the RNA-puzzle toolkit to calculate the INF score. We also calculated the Template Modeling score (TMscore) of the predicted models, which is a well-known similarity metric in computational structural biology for measuring the quality of predicted models and is independent of the size of the target unlike RMSD [83].


$$TMscore=max\left[\frac{1}{L_{target}}\sum_{i=1}^{L_{common}}\frac{1}{1+{\left(\frac{d_{i}}{d_{0}(L_{target})}\right)}^{2}}\right]$$

Here, *L*_*target*_ is the length of the target(native) RNA, and *L*_*common*_ is the number of nucleotides common in the aligned template(model) and target(native) structures. *d*_*i*_ is the distance between *i*^*th*^ pair of aligned nucleotides in the model and native structures, and $d_{0}(L_{target})=0.6*\sqrt{L_{target}-0.5-2.5}$ is a scaling factor which approximates the average distance of corresponding residue pairs of random related RNAs (making TMscore independent of RNA sequence length). TMscore provides a score between 0 and 1, with 1 being an exact match to the native and 0 being the lowest. Generally, a TMscore <0.17 means that the structures are indistinguishable from random pairs, while a score >0.45 suggests that the structures have the same global topological fold [84]. As TMscore gives larger weights to smaller distance errors, it makes the score more sensitive to the global fold similarity unlike RMSD, which is more sensitive to local structural variations. We included an additional metric to measure the fraction of native contacts recovered in the modelled structures [85]. This Native Contact Fraction metric calculates the fraction of residue contacts (a contact is defined as two non-contiguous residues occurring within a distance cut-off of 5 Å) that are also present in the modelled structures. It can simply be calculated by the ratio of contacts that occur in the predicted structure to the contacts in the native structure, i.e., the recall of the native contacts in the modelled structure.

## Results

### Results on the CASP15 dataset

**Fig 1:**
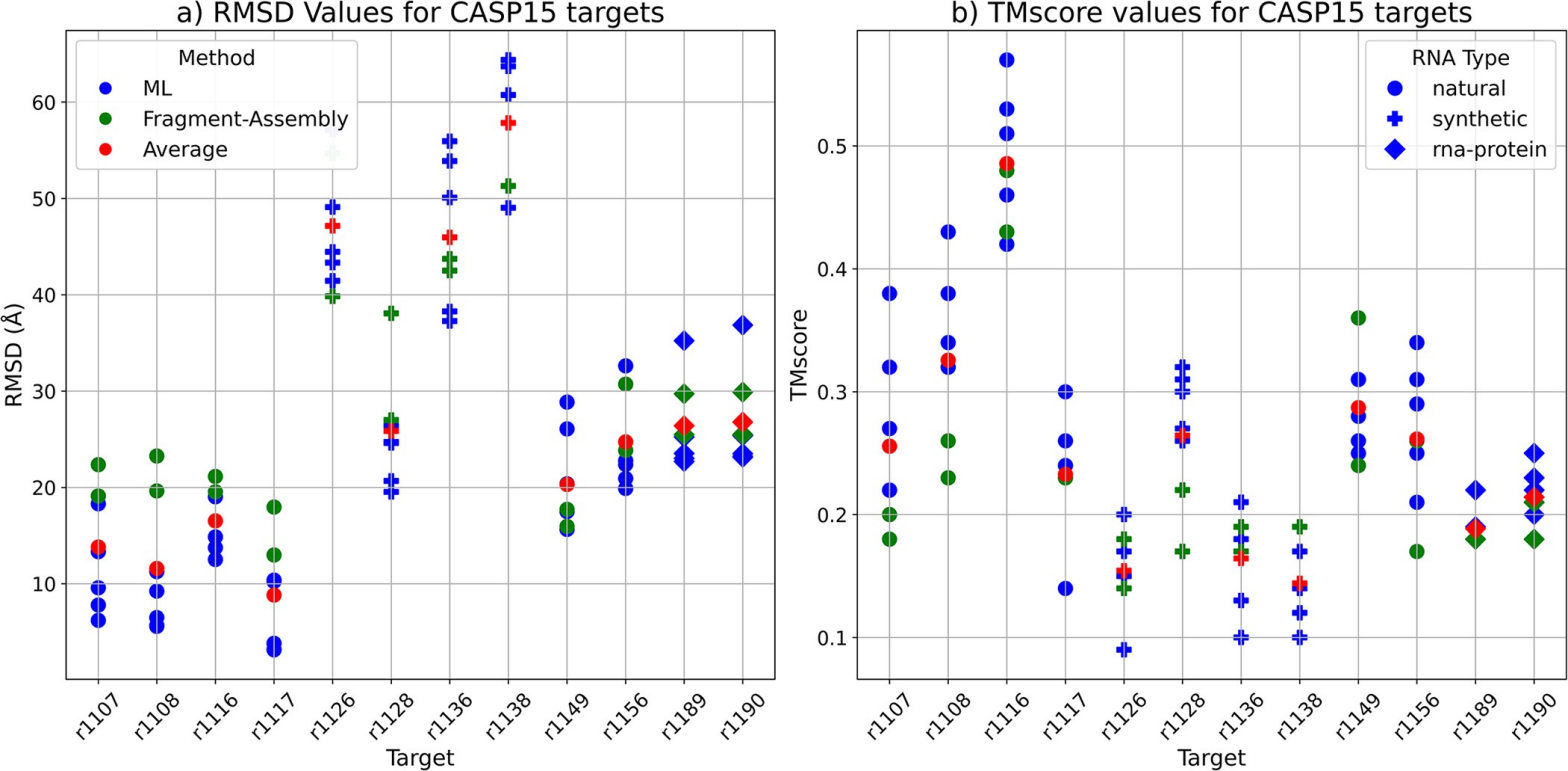
RMSD and TMscore comparison for the RNA targets in the CASP15 dataset. Models predicted by Machine-Learning-based (ML-based) methods are coloured in blue, the ones predicted by Fragment-Assembly-based (FA-based) methods are in green and the average RMSD of all models for each target is in red. The shape of the points is based on the RNA type with circle denoting a natural RNA, + denoting a synthetic RNA and a square denoting an RNA-protein complex **a)** Plot showing the RMSD values in Å for the twelve targets in the CASP15 dataset. For natural RNAs (r1107 and r1108), the ML-based methods (in blue) have much lower RMSD than the average (in red) and the FA-based methods (in green). The best model for each target is the one usually predicted by a ML-method (except for R1126 which is a synthetic target with a length of 363 nucleotides). The average RMSD for most synthetic targets is higher than the natural and RNA-protein complex targets. **b)** Plot showing the TMscores for the predicted models for each target. TMscore for natural targets (r1107 and r1108) is much higher compared to the synthetic and RNA-protein targets. For the natural targets, ML-predicted models have higher TMscore than the average and the FA-predicted models. Model with the best TMscore for each target is one predicted by a ML-based method (except for r1138 which is a very long synthetic RNA of 720 nucleotides).

**Fig 2:**
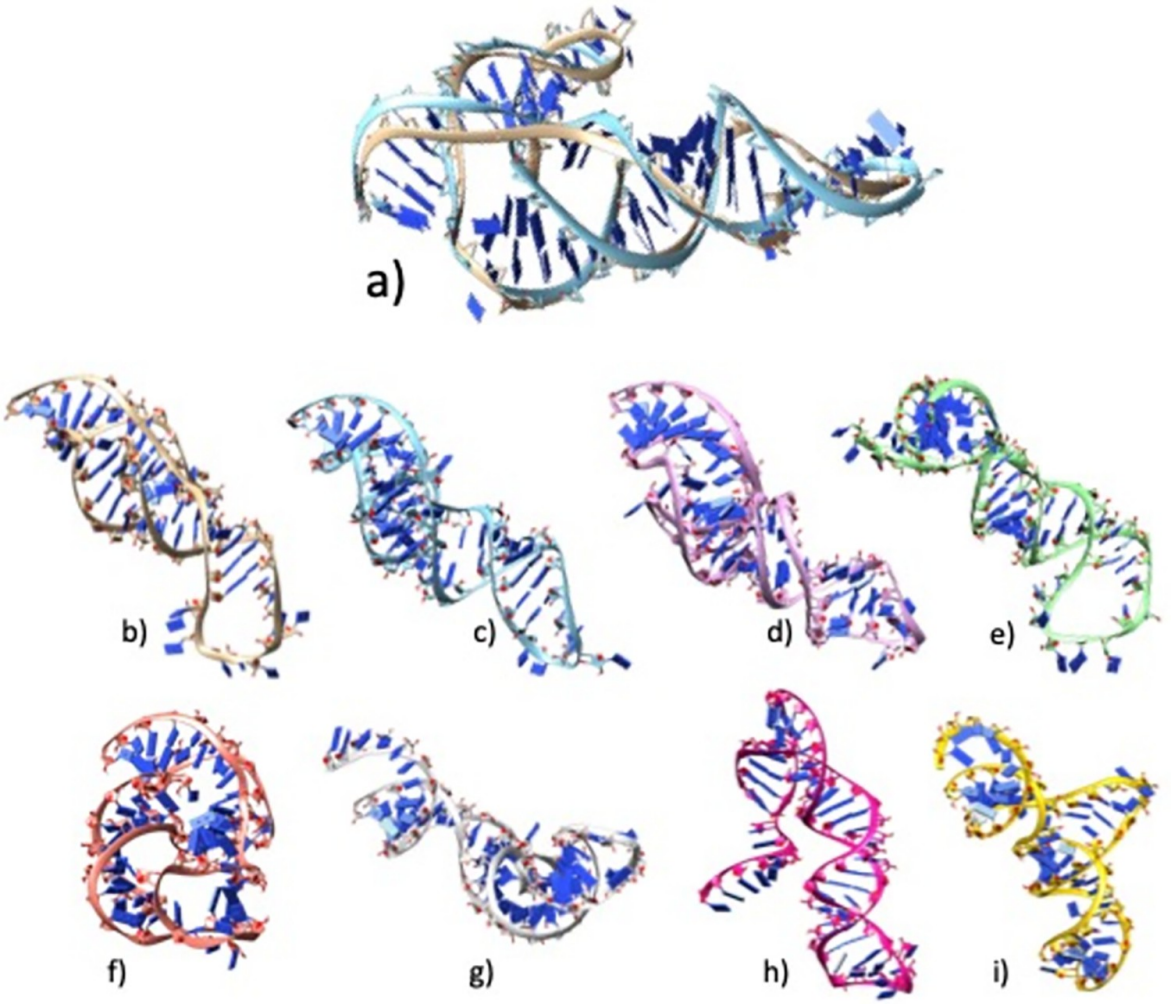
Native and predicted models for CASP target R1107. All the structures were aligned together against the native(b) and then tiled separately to visualize. **a)** Superimposition of native structure (in beige colour) to the best model (DeepFoldRNA, cyan colour); RMSD = 6.19 Å **b)** Native structure **c)** DeepFoldRNA model **d)** RhoFold model; RMSD = 7.79 Å **e)** RosettaFold2NA model; RMSD = 9.58 Å **f)** trRosettaRNA model; RMSD = 13.35 Å **g)** DRFold model; RMSD = 18.30 Å **h)** RNAComposer model; RMSD = 19.12 Å **i)** 3DRNA model; RMSD = 22.54 Å.

**Fig 3:**
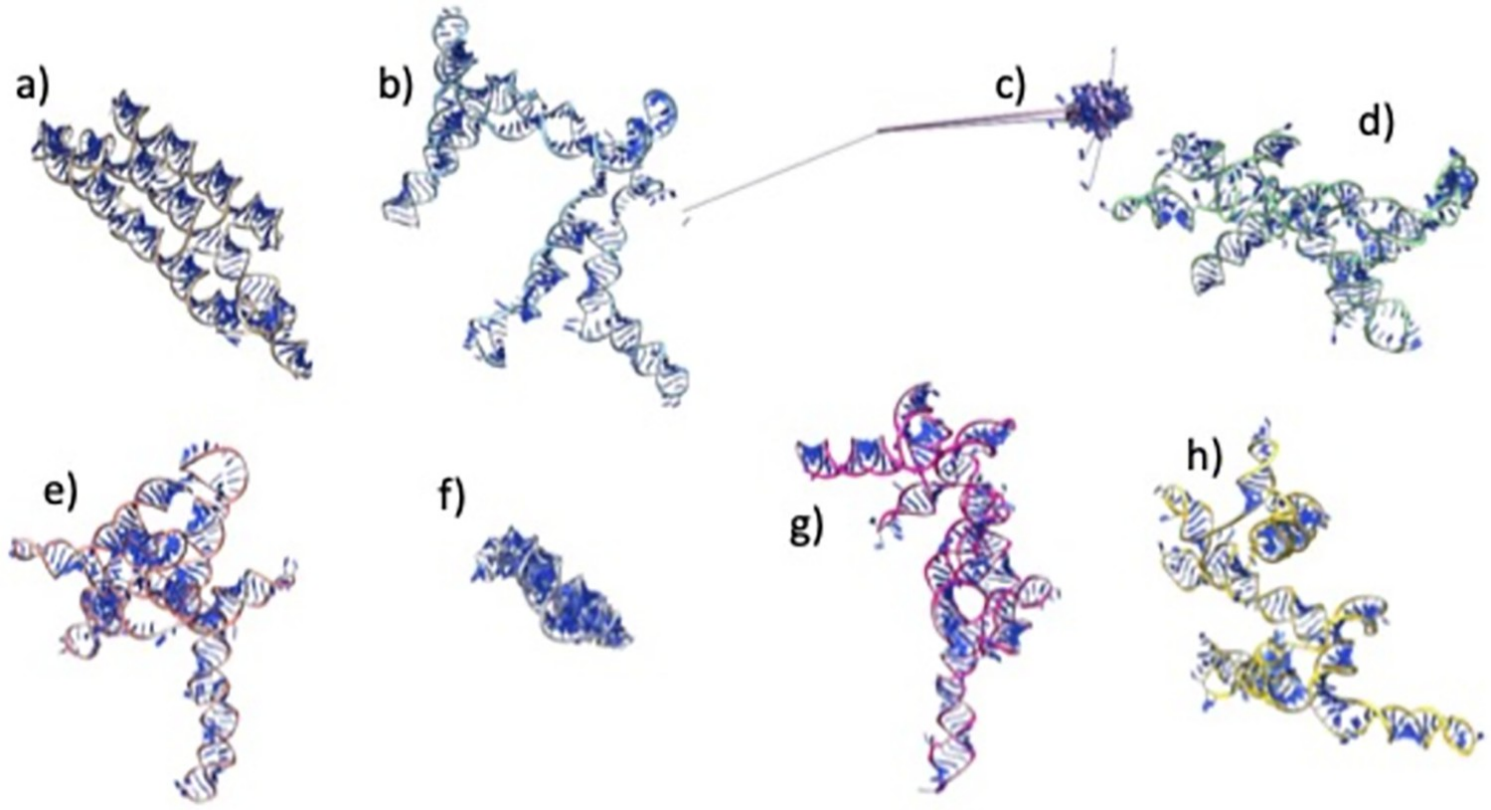
Native and predicted models for CASP target R1136. All the structures were aligned together against the native(a) and then tiled separately to visualize. **a)** Native structure **b)** DeepFoldRNA model; RMSD = 37.26 Å **c)** RhoFold model; RMSD = 55.94 Å **d)** RosettaFold2NA model; RMSD = 53.88 Å **e)** trRosettaRNA model; RMSD = 38.27 Å **f)** DRFold model; RMSD = 50.08 Å **g)** RNAComposer model; RMSD = 42.49 Å **h)** 3dRNA model; RMSD = 43.72 Å

The average RMSD of the DRFold predicted models was the lowest (RMSD = 22.62 Å), while the RosettaFold2NA models had the highest RMSD 29.89 Å overall (Table 5 and Fig 1A). Notably, the best models from the CASP15 methods were much better than the ones in our benchmarking (Table 6). The RMSDs for natural targets (R1107 and R1108) were much lower than that for the other targets for all the methods, and the RMSDs for both the synthetic and RNA-protein complexes were much higher (Fig 1A). Same was true for other metrics as well (Figs 1B and S1 Fig). We selected two targets (R1107 and R1136) as examples for visualizing good and poor quality predictions by showing the 3D structural alignment of their models with the native structure. The models predicted by most methods for R1107 are predicted reasonably well and we see a good alignment with the native structure (Fig 2). All the models for R1136 were of poor quality as it is a very long synthetic RNA and therefore the structural alignment wasn’t good at all (Fig 3).

**Table 5:** Targets from the RNA3DB dataset. ^a^E = Cryo-electron microscopy, ^b^X = X-Ray crystallography. These targets were compiled from the RNA3DB dataset and are recommended as a test det for ML-based RNA prediction methods. These targets are all published after the ML-based RNA-prediction methods and aren’t part of the their training set. The targets are also non-homologous to existing RNA sequences in the PDB database and don’t belong to any previously known RFam families in the PDB. These so-called orphan RNA’s will serve as a stringent dataset for the prediction methods.

Name	Type	Length	Experimental Method (resol Å)	RNA Type	Difficulty
**8ane_r**	RNA-protein	52	E^a^ (3.20)	type I-G CRISPR effector	synthetic
**8d1v_j**	RNA-protein	25	E (2.80)	guide RNA	natural
**8d29_r**	RNA-protein	26	X^b^ (1.81)	theophylline aptamer	synthetic
**8d4a_b**	RNA-protein	31	E (2.74)	Cas12a2 quaternary complex	synthetic
**8do6_j**	RNA-protein	22	E (3.10)	Cas10-Csm bound to target RNA	natural
**8e0f_d**	RNA-protein	26	X (2.70)	dsRNA (ADAR2-RD)	natural
**8ewg_b**	RNA-protein	42	E (2.90)	riboendonclease	natural
**8f0n_b**	RNA dimer	37	X (2.85)	Wobble Beetroot (A16U-U38G) dimer	synthetic
**8ff5_m**	RNA-protein	46	E (3.13)	type I-B CAST system	synthetic
**8ffy_c**	RNA-protein	31	E (3.60)	mt-tRNA(UGA-TL)	natural
**8gkh_w**	RNA-protein	59	E (2.70)	omega RNA	natural
**8gxb_b**	RNA-protein	43	X(2.15)	NAD+ -II riboswitch	natural
**8h7q_d**	RNA-protein	25	E (3.80)	Synechocystis sp. PCC6714 Cascade	synthetic
**8hb8_a**	RNA	37	X(2.30)	NAD-II riboswitch (single strand)	natural
**8hud_b**	RNA-protein	51	E (3.43)	EvCas9-sgRNA-target DNA complex	synthetic
**8ibw_d**	RNA-protein	29	E (3.60)	R2 with 3’UTR	natural
**8j1q_e**	RNA-protein	33	E (3.30)	SARS CoV-2 RBD and Aptamer complex	synthetic
**8sqj_p**	RNA-protein	29	E (3.06)	RNA-nsp9 bound to SARS-CoV-2	natural
**8sqj_t**	RNA-protein	32	E (3.06)	RNA-nsp9 bound to SARS-CoV-2	natural
**8upt_a**	RNA	47	E (2.80)	tRNAPyl	Natural

**Table 6:** Comparison of RMSD values of the predicted models by the various methods to native structure for targets in the CASP15 dataset. DRFold has the lowest average RMSD of 22.62 Å, which is slightly better than that of DeepFoldRNA (24.49 Å). ‘best_rmsd_ours’ column denotes the rmsd of the best model predicted by our benchmarking methods and ‘best_rmsd_casp’ column contains the rmsd of the best model from all the methods participating in CASP15. Looking at the ‘best_rmsd_ours’ column clearly shows that we are only able to predict models of reasonably well quality for only three of the seven targets(r1107, r1108 and r1117).

	df	drfold	rf	rhofold	rnac	trr	3drna	best_ rmsd_ours	best_ rmsd_casp
**r1107**	6.19	18.3	9.58	7.79	19.12	13.33	22.36	6.19	4.5
**r1108**	5.67	9.24	11.25	6.49	19.64	5.57	23.25	5.67	4.5
**r1116**	14.87	13.74	19.03	12.52	19.55	14.84	21.14	12.52	4.8
**r1117**	10.38	3.14	10.23	3.82	17.95	3.2	12.98	3.14	2.01
**r1126**	49.09	41.45	57.12	43.32	54.68	44.45	39.84	39.84	4.3
**r1128**	24.71	26.39	24.6	20.67	26.98	19.52	38.05	19.52	7.2
**r1136**	37.26	50.08	53.88	55.94	42.49	38.27	43.72	38.27	7.8
**r1138**	60.74	NA	63.71	64.38	NA	49.02	51.28	49.02	16.6
**r1149**	15.64	17.49	26.07	28.86	15.95	20.37	17.72	15.64	6.88
**r1156**	22.37	22.77	32.61	20.92	30.71	19.89	23.84	19.89	5.37
**r1189**	23.51	23.01	25.25	22.68	25.46	35.22	29.72	23.01	16
**r1190**	23.5	23.22	25.38	23.13	25.48	36.85	29.89	23.13	15.9
**Average**	**24.49**	**22.62**	**29.89**	**25.88**	**27.09**	**25.04**	**29.48**		

df: DeepFoldRNA, drfold: DRFold, rf: RosettaFold2NA, rhofold: RhoFold, rnac: RNAComposer, trr: trRosettaRNA, 3drna: 3dRNA

### Results on the new dataset

**Fig 4:**
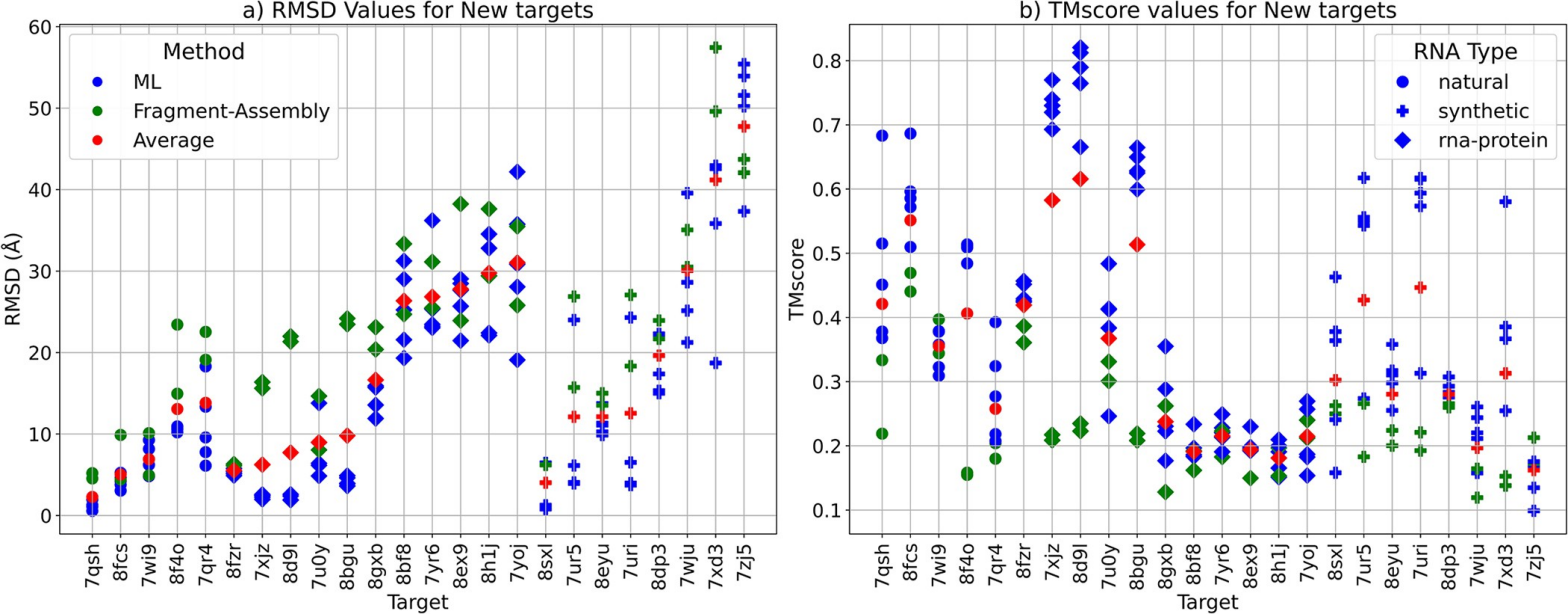
RMSD and TMscore comparison for the RNA targets in the New dataset. Models predicted by Machine-Learning-based (ML-based) methods are coloured in blue, the ones predicted by Fragment-Assembly-based (FA-based) methods are in green and the average RMSD of all models for each target is in red. The shape of the points is based on the RNA type with circle denoting a natural RNA, + denoting a synthetic RNA and a square denoting an RNA-protein complex **a)** Plot showing the RMSD values in Å for the targets in the New dataset. For most targets, the ML-based methods (in blue) have much lower RMSD than the average (in red) and the FA-based methods (in green). The average RMSD for most synthetic targets is higher than the natural and RNA-protein complex targets. **b)** Plot showing the TMscores for the predicted models for each target. TMscore for almost all targets for ML-methods (in blue) is higher compared to the Average(in red) and FA-based methods (in green). Model with the best TMscore for each target is one predicted by a ML-based method. The average TMscore for most synthetic targets is higher than the natural and RNA-protein complex targets.

On this dataset, the DeepFoldRNA has the lowest average RMSD overall, with DRFold being the close second (Table 7). The fragment-assembly based methods had the highest RMSD overall with the worst prediction performance (Table 6 and Fig 4). We also observed that generally the natural targets had lower RMSD (and higher TMscore) when compared to the synthetic and RNA-protein complex targets (Fig 4). The ML-based methods again had a better performance than the FA-based methods with the best model (based on RMSD and TMscore) for each target being always the one predicted by a ML-based method. This was true for other metrics as well (S2 Fig).

**Table 7:** Comparison of RMSD values of the predicted models to the native structure for targets in the newly compiled dataset. DeepFoldRNA(df) has the lowest average RMSD (12.84 Å) on this dataset. RMSD for the fragment-assembly-based methods (3DRNA and RNAComposer) is much higher than that of the deep-learning-based methods.

Target	df	drfold	rf	rhofold	rnac	trr	3drna
**7yoj**	28.07	35.75	42.18	30.85	25.79	19.1	35.48
**7uri**	3.73	3.92	24.3	3.99	27.07	6.52	18.34
**7qr4**	6.12	18.3	9.58	7.78	19.12	13.35	22.54
**8h1j**	22.11	29.43	34.54	22.41	29.41	32.81	37.63
**8dp3**	21.79	15.32	15.01	22.3	21.68	17.38	23.94
**7wju**	28.62	21.24	39.57	25.14	35.06	30.31	30.5
**8bgu**	3.95	3.62	4.89	3.88	23.47	4.62	24.19
**7xjz**	2.47	2.58	2.25	1.99	15.61	2.44	16.38
**8ex9**	27.66	29.04	25.69	21.46	23.92	28.48	38.24
**7ur5**	4.06	4.04	24	3.93	26.88	6.14	15.72
**7xd3**	18.72	NA	42.94	35.84	57.44	42.57	49.6
**7wi9**	4.84	8.17	6.22	4.83	4.93	9.26	10.11
**8f4o**	10.57	10.65	10.94	10.21	14.95	10.75	23.43
**8d9l**	2.39	1.91	2.6	1.89	21.34	1.92	22
**8gxb**	13.57	15.9	15.72	11.91	23.12	15.91	20.38
**8sxl**	0.8	0.84	1.26	6.41	6.21	6.46	6.24
**7u0y**	6.41	6.16	8.98	13.8	14.66	4.86	8.05
**8eyu**	9.87	11.09	13.75	10.28	13.54	11.34	15.01
**8bf8**	21.58	31.25	25.24	19.32	24.7	29.02	33.35
**7yr6**	23.45	23.22	25.36	23.04	25.48	36.21	31.13
**7zj5**	37.33	50.2	53.93	55.41	42.07	51.56	43.72
**7qsh**	1.28	1.08	0.58	1.28	4.55	1.98	5.2
**8fcs**	3.78	3.06	5.24	4.23	9.89	4.65	4.43
**8fzr**	5.13	5.17	5.39	4.9	6.32	5.78	6.11
**Average**	**12.84**	**14.43**	**18.34**	**14.46**	**21.55**	**16.39**	**22.57**

df: DeepFoldRNA, drfold: DRFold, rf: RosettaFold2NA, rhofold: RhoFold, rnac: RNAComposer, trr: trRosettaRNA, 3drna: 3dRNA

### Results on the RNA-Puzzles dataset

**Fig 5:**
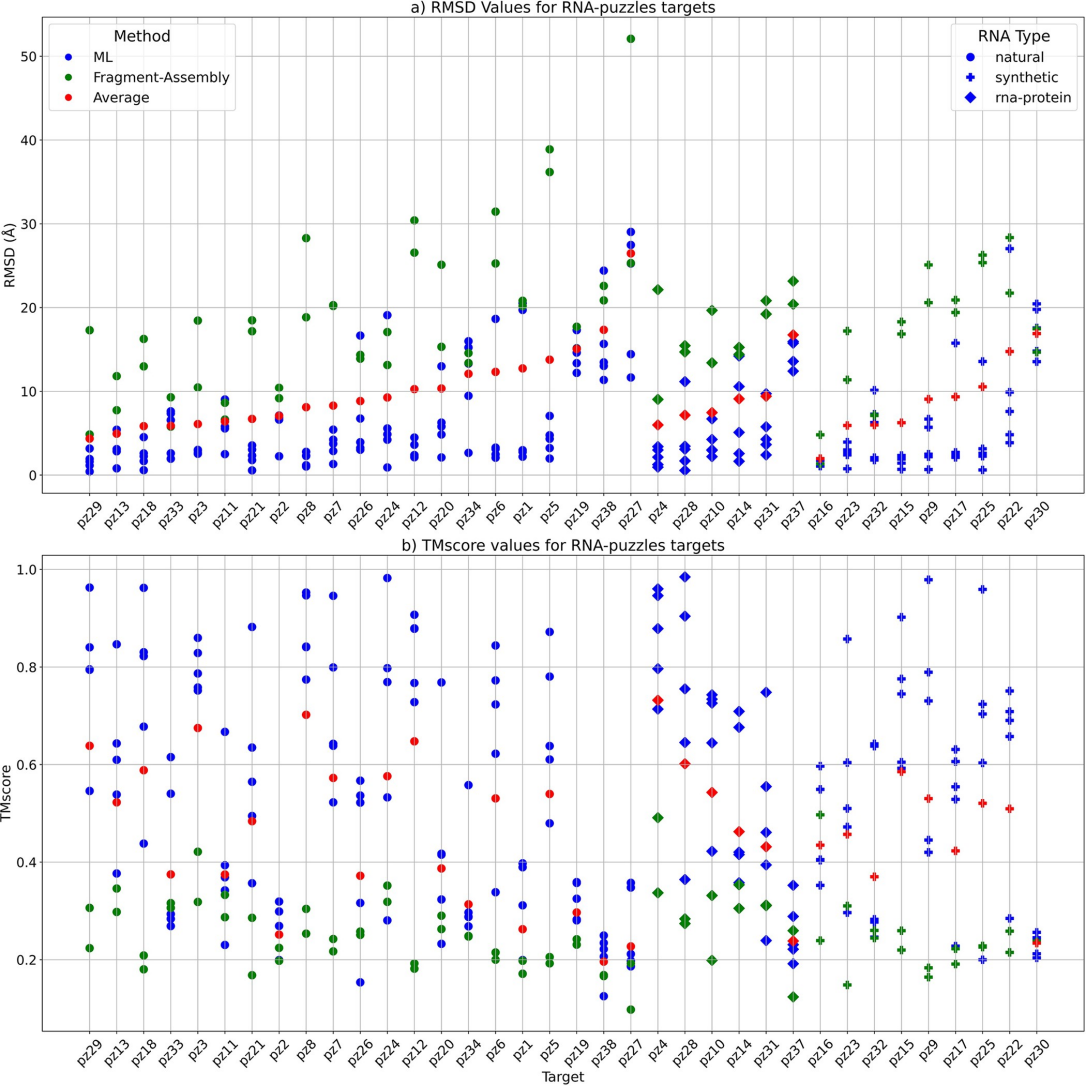
RMSD and TMscore comparison for the RNA targets in the RNA-puzzles dataset. Models predicted by Machine-Learning-based (ML-based) methods are coloured in blue, the ones predicted by Fragment-Assembly-based (FA-based) methods are in green and the average RMSD of all models for each target is in red. The shape of the points is based on the RNA type with circle denoting a natural RNA, + denoting a synthetic RNA and a square denoting an RNA-protein complex. On average, the performance of most methods on this dataset is much better than on CASP15 or the New dataset, possibly because many targets might have been part of the training set of the ML-methods and also many homologous structures for these targets are available in the PDB. The ML-based methods have the best quality models (low RMSD and High TMscore) and the FA-based methods have the lowest quality models for most targets in this dataset **a)** Plot showing the RMSD values in Å for the targets in the CASP dataset. For most targets, the ML-based methods (in blue) have much lower RMSD than the average (in red) and the FA-based methods (in green). **b)** Plot showing the TMscores for the predicted models for each target. TMscore for almost all targets for ML-methods (in blue) is higher compared to the Average(in red) and FA-based methods (in green). Model with the best TMscore for each target is always the one predicted by a ML-based method.

The average results on this dataset are much better than the other datasets. DRFold has the best performance (lowest average RMSD) on this dataset followed closely behind by DeepFoldRNA as the second best. The other ML-based methods also have a comparatively lower average RMSDs on this dataset compared to the others. However, the performance of the FA-based methods lags far behind the ML-based methods (Table 8). We also see that on pz34, pz37 and pz38 targets (more challenging as they were definitely not included in the training set of ML-based methods), the performance of our methods was much worse (except for RhoFold which has 2.65 Å model for pz34 because it was published in early 2022 and possibly includes it in its training set). Surprisingly, unlike the other datasets we didn’t see a much better average performance for natural targets compared to synthetic and RNA-complex targets on this dataset (Fig 5). The performance of the methods based on other metrics was also a lot better on this dataset (S3 Fig).

**Table 8:** Comparison of RMSD values (in Å) of the predicted models to the native structure for targets in the newly compiled dataset. DRFold has the lowest average RMSD (5.40 Å) on this dataset. RMSD for the fragment-assembly-based methods (3DRNA and RNAComposer) is much higher than that of the deep-learning-based methods.

Target	df	drfold	rf	rhofold	rnac	trr	3drna
**pz1**	2.97	2.2	20.52	19.72	20.18	2.76	20.81
**pz2**	2.25	6.62	NA	6.84	9.18	7.11	10.42
**pz3**	2.55	2.64	2.63	3.01	18.44	2.92	10.46
**pz4**	2.99	1.25	0.95	3.4	22.13	2.14	9.04
**pz5**	4.76	1.98	7.07	4.33	38.89	3.23	36.17
**pz6**	3.12	2.07	2.47	3.28	31.45	18.64	25.27
**pz7**	2.87	1.32	4.22	3.74	20.19	5.43	20.28
**pz8**	2.34	1.18	1.02	2.78	18.84	2.28	28.29
**pz9**	6.69	2.2	0.66	5.7	25.09	2.49	20.58
**pz10**	2.95	4.25	2.21	6.71	19.66	3.02	13.41
**pz11**	5.8	6.63	9.03	5.57	8.63	2.5	6.62
**pz12**	3.62	2.13	2.39	4.48	30.4	2.24	26.56
**pz13**	2.84	3.1	0.8	5.41	11.82	2.87	7.75
**pz14**	5.1	1.65	10.57	14.22	14.4	2.57	15.25
**pz15**	2.34	1.43	0.68	2.22	16.84	1.9	18.29
**pz16**	1.79	1.07	1.13	1.83	1.35	1.76	4.8
**pz17**	2.34	2.71	15.75	2.22	20.9	2.11	19.41
**pz18**	2.59	1.67	0.59	4.53	12.98	2.25	16.25
**pz19**	12.2	14.62	17.31	13.36	17.71	15.14	15.09
**pz20**	4.84	6.27	12.99	5.8	15.31	2.11	25.11
**pz21**	3.05	2.33	0.56	3.54	18.48	1.79	17.18
**pz22**	7.59	3.86	9.9	27.03	28.35	4.81	21.72
**pz23**	2.99	2.75	0.75	3.94	11.37	2.4	17.2
**pz24**	5.57	4.87	0.91	19.09	13.15	4.22	17.08
**pz25**	2.6	2.25	0.61	13.56	26.26	3.14	25.35
**pz26**	16.66	3.25	3.03	6.75	13.9	3.92	14.35
**pz27**	14.44	29.03	25.27	27.48	52.08	11.66	25.31
**pz28**	3.44	1.69	0.55	11.16	14.7	3.08	15.46
**pz29**	1.92	1.16	0.42	3.17	17.29	1.62	4.85
**pz30**	13.54	14.8	19.78	17.56	14.68	20.44	17.46
**pz31**	3.64	5.77	9.72	4.28	19.22	2.4	20.82
**pz32**	2.07	7.26	10.12	1.79	7.18	6.27	7.11
**pz33**	2.6	7.36	6.56	7.62	5.79	1.95	9.29
**pz34**	15.24	9.47	13.32	2.65	14.57	15.98	13.39
**pz37**	13.58	15.94	15.75	12.41	23.15	15.94	20.39
**pz38**	11.36	15.66	24.41	13.46	22.58	13.04	20.85
**Average**	**5.48**	**5.40**	**7.28**	**8.18**	**18.81**	**5.50**	**17.16**

df: DeepFoldRNA, drfold: DRFold, rf: RosettaFold2NA, rhofold: RhoFold, rnac: RNAComposer, trr: trRosettaRNA, 3drna: 3dRNA

### Combined results

**Fig 6:**
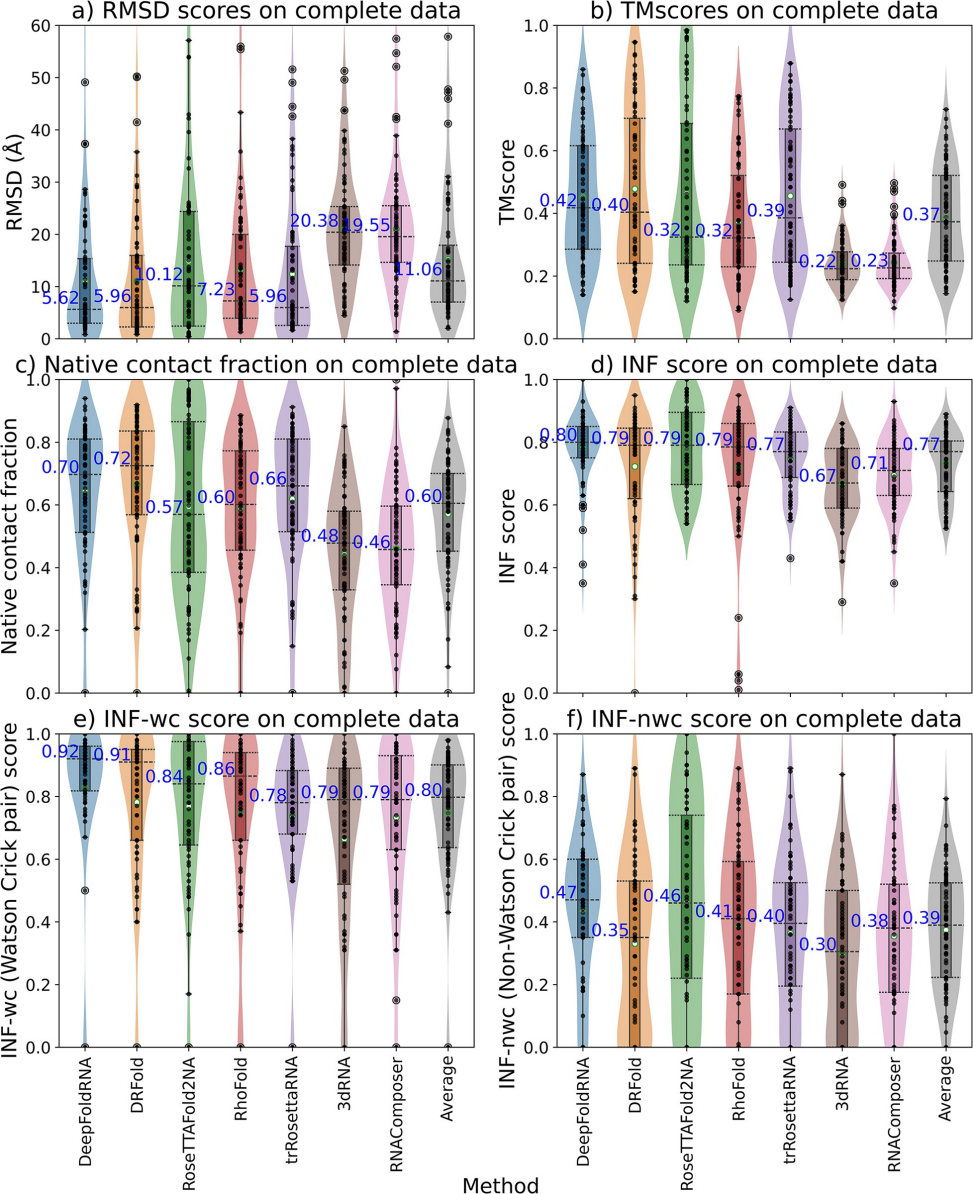
Box and violin plots showing the comparison of all the methods on the combined dataset across multiple metrics. The methods are on the X-axis and the metrics are on the Y-axis. The Average plot (in grey) is the average of the models predicted by all the methods for a particular target. The median values are labelled with blue text and the whiskers denote the interquartile range **a)** RMSD distribution of the predicted models by various methods. DeepFoldRNA has the lowest median RMSD (5.62 Å). **b)** TMscore distribution for the various methods. DeepFoldRNA and DRFold have the highest median TMscore **c)** Native contact fraction (ncf) of the predicted models by the various methods. DRFold has the highest ncf of 0.72. **d)** INF score for the various methods. DeepFoldRNA has the highest median INF score (0.80) **e)** INF-wc score (Watson-Crick pairs) for the various methods. DeepFoldRNA has the highest median score of 0.92. Most methods have a really good INF-wc score (> = 0.8 for most methods) indicating that the canonical Watson-Crick pairs are predicted quite accurately by most methods. Interestingly, for the first time for a metric, a ML-based method i.e. trRosettaRNA has a median score lower than the medians of the Average prediction or FA-based methods (3DRNA, RNAcomposer). This could be because the secondary structure prediction method used by trRosettaRNA might not be as accurate as others. **f)** INF-nwc score (non-Watson-Crick pairs) for the various methods. DeepFoldRNA has the highest median RMSD of 0.47. None of the methods even have a median score higher than 0.5 indicating that none of the methods are very good at predicting non-canonical base pairing. Interestingly, again this time a ML-based method (DRFold) has a lower median score than the Average as well as median score of RNAComposer (an FA-based method). Usually, DRFold has been the second-best method and close to DeepFoldRNA on most metrics, so this discrepancy might be explained because of its non-reliance on MSA as input to predict the structure, as all other ML-based methods use MSA and they are able to predict non-Watson-Crick pairs more accurately.

We finally looked at the performance of all the methods on the combined dataset, i.e., CASP15+New+RNA-Puzzles to get an idea about the generalized performance of the methods (Fig 6). The performance was compared using several metrics including RMSD, TMscore, Native contact fraction (Ncf), Interaction network fidelity (INF) for all pairs, INF-wc (INF for Watson-Crick pairs), and INF-nwc (INF for non-Watson-Crick pairs). DeepFoldRNA had the lowest median RMSD overall, while 3DRNA had the highest. We also included average prediction as an additional comparison method, which is basically the average of the metric score of the model predicted by all of the seven methods for each target. We observed that all the five deep-learning methods have a better performance than the average prediction and the two fragment-assembly-based methods have a worse performance than the average prediction based on RMSD (Fig 6A), TMscore (Fig 6B), Ncf (Fig 6C) and INF score (Fig 6D). The INF score is essentially a metric quantifying how accurately is the base-pairing of the nucleotides predicted. The INF-wc and INF-nwc score are the only two metrics where any of the ML-based methods have a worse median than any of the FA-based methods (Fig 6E and 6F). Although most of the methods are able to predict the Watson-Crick pairs quite well (Fig 6E), none of the methods are good in predicting non-canonical base pairs as the median RMSD of even the best method for INF-nwc score is only 0.47. This is a known limitation in the field of RNA secondary structure prediction [86–88], but even the end-to-end tools (RosettaFold2NA, RhoFold) which predict these base-pair contacts implicitly inside the neural network without relying on an external tool for ss prediction or ss input, have a low INF-nwc score.

**Fig 7:**
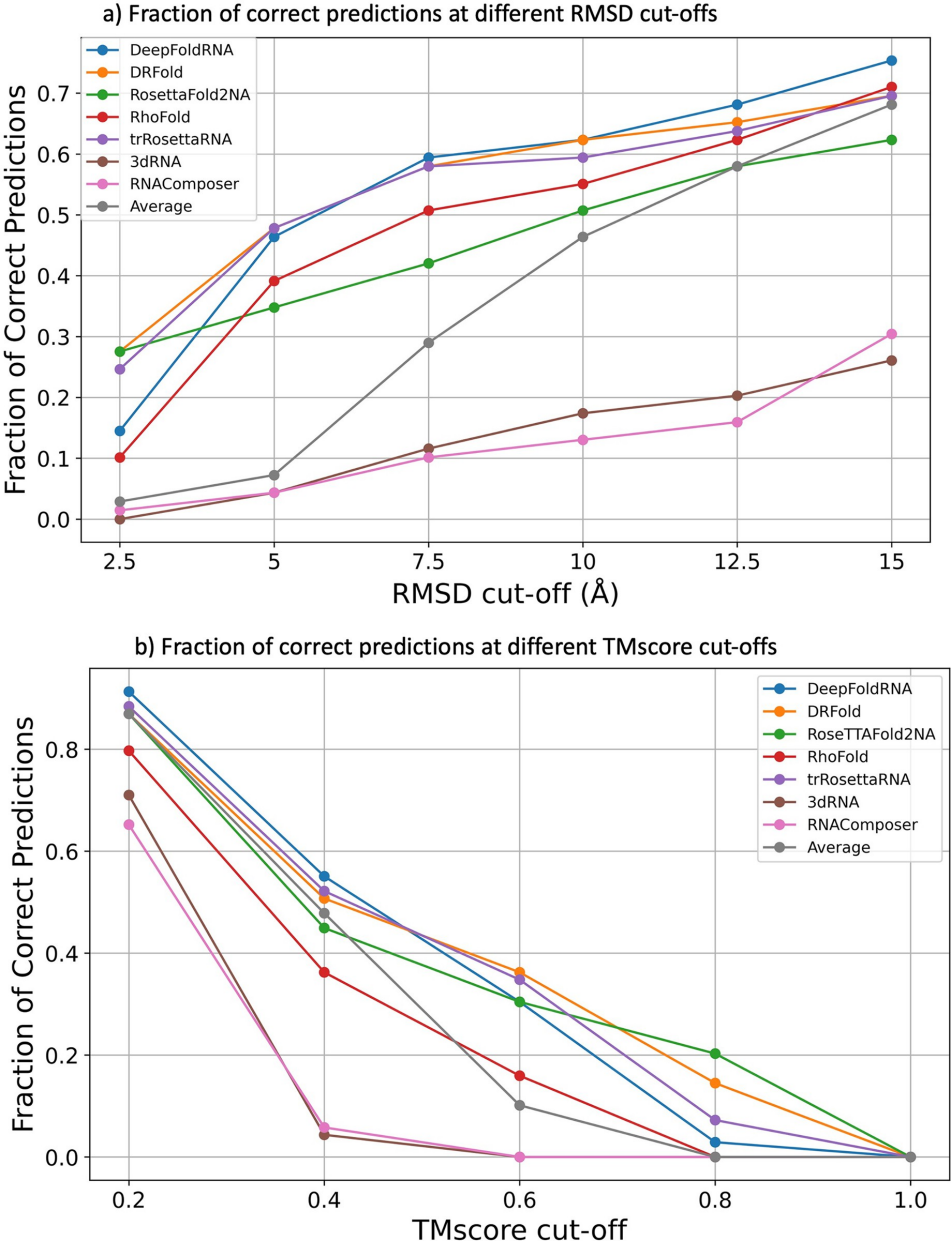
Plots showing the performance of various RNA structure prediction methods at different RMSD and TMscore cut-offs. **a)** At a RMSD cut-off of 5 Å, DeepFoldRNA, DRFold and trRosettaRNA are able to predict 50% of the targets correctly, which increases to 70–75% on increasing the cut-off to 15 Å. However, RNAComposer and 3DRNA are only able to predict 5% of the targets correctly at 5 Å cut-off and even after increasing the RMSD cut-off for correct predictions to 15 Å, they are only able to predict around 30% of the targets correctly. **b)** At a TMscore cut-off of 0.4 most ML-based methods are able to predict ~50% targets correctly, while FA-based methods are only able to predict <5% targets correctly. On applying a more stringent cut-off of 0.6 the % of correct predictions for the ML-methods drops below 40% while FA-methods aren’t even able to predict a single model with a TMscore higher than 0.6.

We can also look at the performance of all the methods by looking at the fraction of correctly predicted targets out of all targets. A predicted model is termed as a correct prediction, if the RMSD/TMscore of the predicted model is less/more than a certain RMSD/TMscore cut-off. We used RMSD cut-offs of 2.5 Å (almost near-native predictions) to 15 Å with intervals of 2.5 Å and TMscore cut-offs of 0.2 to 1. At 5 Å cut-off, DeepFoldRNA, DRFold and trRosettaRNA had close to 50% correct predictions (32/65), whereas RosettaFold2NA had 37% (24/65) and RhoFold had 40% (26/65). For the non-ML methods, they are only able to only predict less than 5% of the targets (3/65) correctly at a cut-off of 5 Å and it only increases to around 30% (17/65 for 3dRNA and 21/65 for RNAComposer) on increasing the cut-off to 15 Å (Fig 7A). Similar results were seen when we looked at different TMscore cut-offs with DL-based methods being much better (DeepFoldRNA being the best) than the FA-based methods overall (Fig 7B).

### RMSD Z-Score analysis

The Z-score tells us how much better or worse a prediction is compared to the average prediction. We calculated the Z-score of all the methods for each target and then looked at the average Z-score for all the targets. A Z-score < 0 indicates that the prediction is better than the average prediction (lower RMSD compared to the average), while a Z-score > 0 indicates a worse prediction than the average (higher RMSD than average).

**Fig 8:**
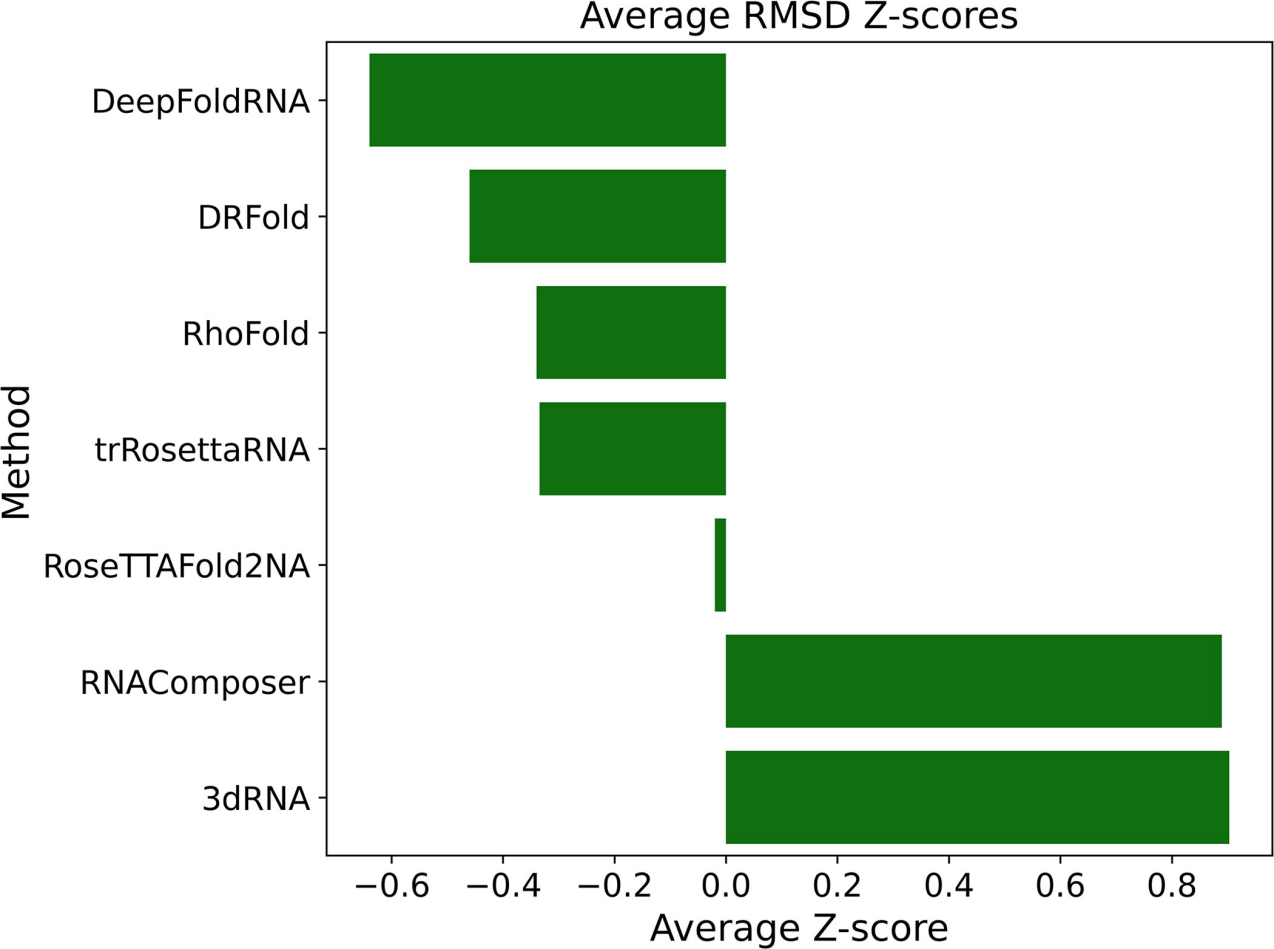
Plot showing the average Z-score of the RMSDs of the predicted structure. A Z-score < 0 indicates that the prediction is better than the average and Z-score > 0 indicates that the prediction is worse than the average. All the machine-learning-based methods have a Z-score < 0 the two fragment-assembly-based methods have a Z-score > 0. DeepFoldRNA has the lowest Z-score, which indicates that it’s predicted models have the lowest RMSD compared to the average prediction.

All the ML-based methods had a Z-score of less than 0 (better than the average prediction), with DeepFoldRNA having the lowest mean Z-score followed by DRFold (Fig 8). The FA-based methods (RNAComposer and 3dRNA) had a Z-score of higher than zero indicating that the predicted models by these methods were worse than the average predictions (higher RMSD). We can also use the Z-score to categorise a prediction as invalid if it has a lot higher RMSD compared to the average. We used a Z-score cut-off of 2 to term a predicted model as invalid (RMSD of the predicted model is two standard-deviations higher than the average prediction). DeepFoldRNA, DRFold, RoseTTAFold2NA had 0 invalid predictions, while trRosettaRNA had 1, and 3dRNA and RNAComposer had 3 invalid predictions each.

### Analysis of method performance based on target difficulty

**Fig 9:**
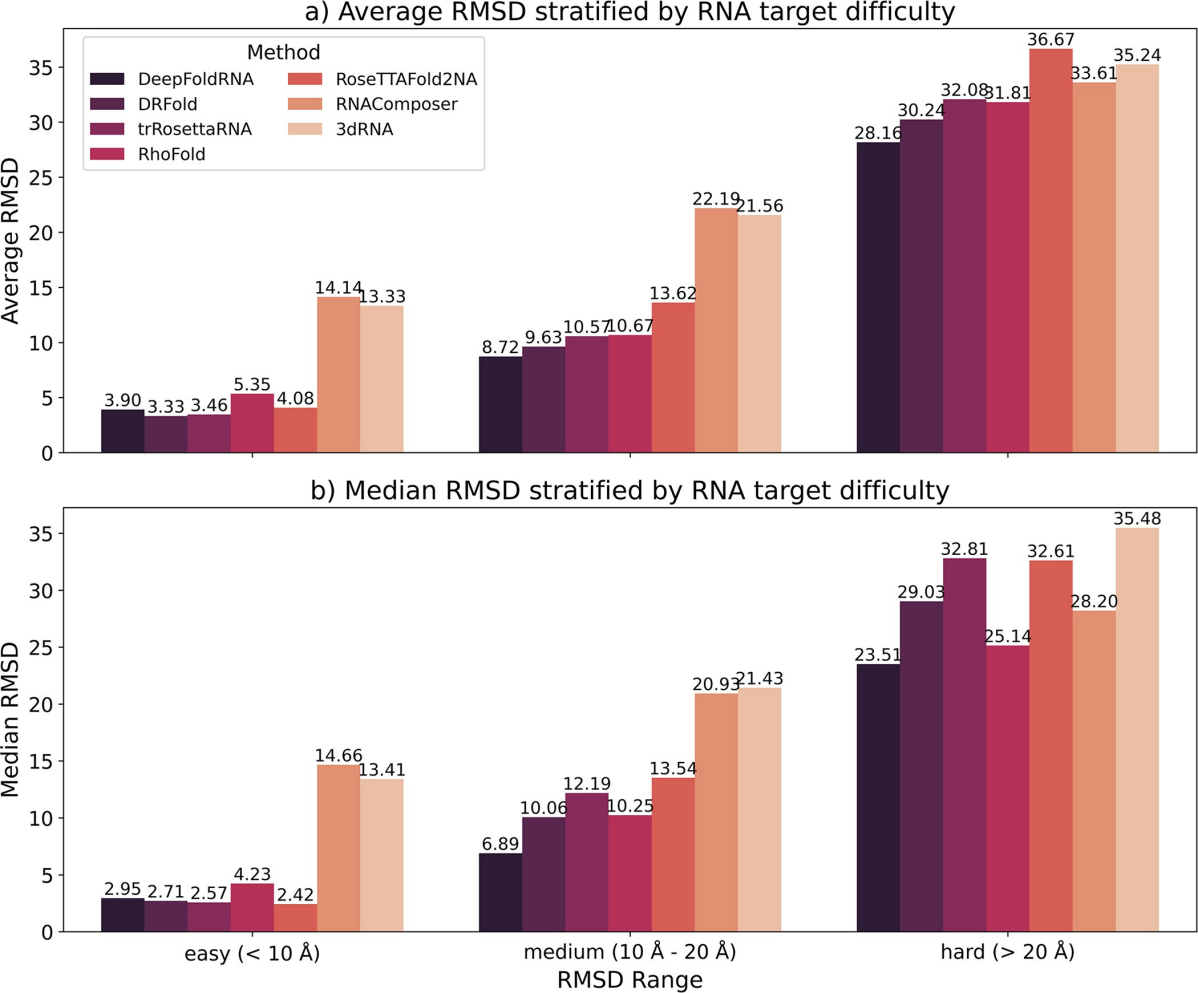
Barplots showing the Mean and Median RMSD of the predicted models by each method depending on the target RNA difficulty. The RNA target difficulty is shown on the X-axis and the Average/Median RMSD is shown on the y-axis. The RNA targets were stratified based on the average of RMSD of all predicted models: easy (average RMSD < 10 Å), medium (average RMSD between 10 Å and 20 Å), and hard (average RMSD > 20 Å). **a)** Mean RMSD for all the methods stratified by RNA target difficulty. **b)** Median RMSD for all the methods stratified by RNA target difficulty.

We analysed the performance of the different method (in terms of RMSD) based on the difficulty of the RNA targets. We defined three different categories of target-difficulty based on the average RMSD of all the predicted models for each target: easy (average RMSD < 10 Å), medium (average RMSD between 10 Å and 20 Å), and hard (average RMSD > 20 Å). We then grouped all the RNA targets into these categories and did a stratified analysis of the method performance (mean and median RMSD) for all the methods. For easy targets, DRFold has the lowest mean RMSD closely followed by DeepFoldRNA. For medium and hard targets, DeepFoldRNA has the lowest mean RMSD (Fig 9A). We observed similar results for the median RMSD as well (Fig 9B).

### Pairwise comparison of different methods

**Fig 10:**
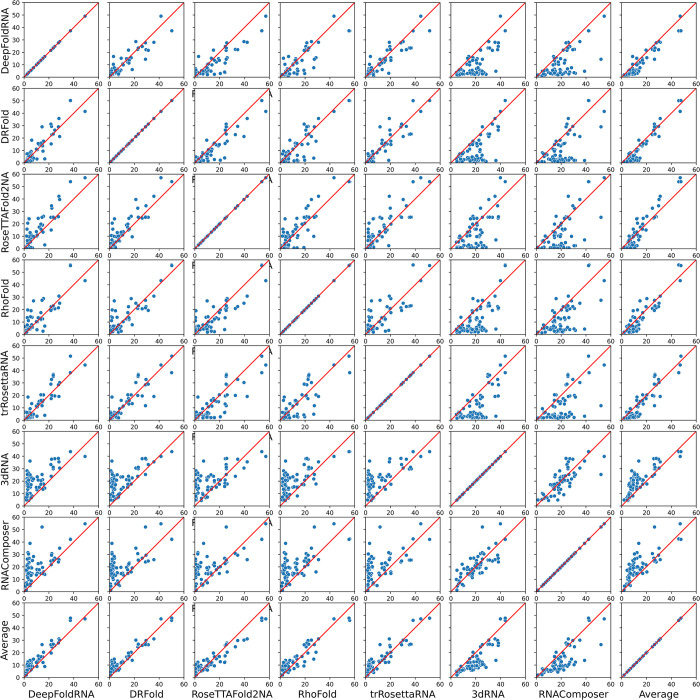
Scatterplot showing the performance comparison of each method against every other method on all the targets. If a point lies on the red-coloured x = y line, it indicates that the RMSD of the predicted model from both the methods is exactly the same i.e. they have similar prediction performance for that target. Points above that line indicate a higher RMSD for the model predicted by the method on the y-axis (i.e. method on the x-axis is better) and points below that line indicate vice-versa. Most of the ML-based methods have a better performance than the average prediction (last row of plots), while the FA-based methods are much worse than the average prediction (Average vs 3dRNA and Average vs RNAComposer plots in the last row). When compared against all other methods using the RMSDs of the predicted models, DeepFoldRNA is the best method followed by DRFold.

We created scatter plots of the RMSD of the predicted models for a pairwise comparison of each method against all other methods. In total we created 8x8 scatter plots (5 ML-based, 2 FA-based and one Average prediction). We observed that generally the ML-based methods have a better performance than the Average prediction and the two FA-based methods (Fig 10). Overall the DeepFoldRNA-predicted models have the best accuracy (in RMSD) when compared against models from all other methods. We also created similar scatterplots for all vs all comparison of the methods based on TMscore and there also DeepFoldRNA turned out to be the best method (S4 Fig).

**Fig 11:**
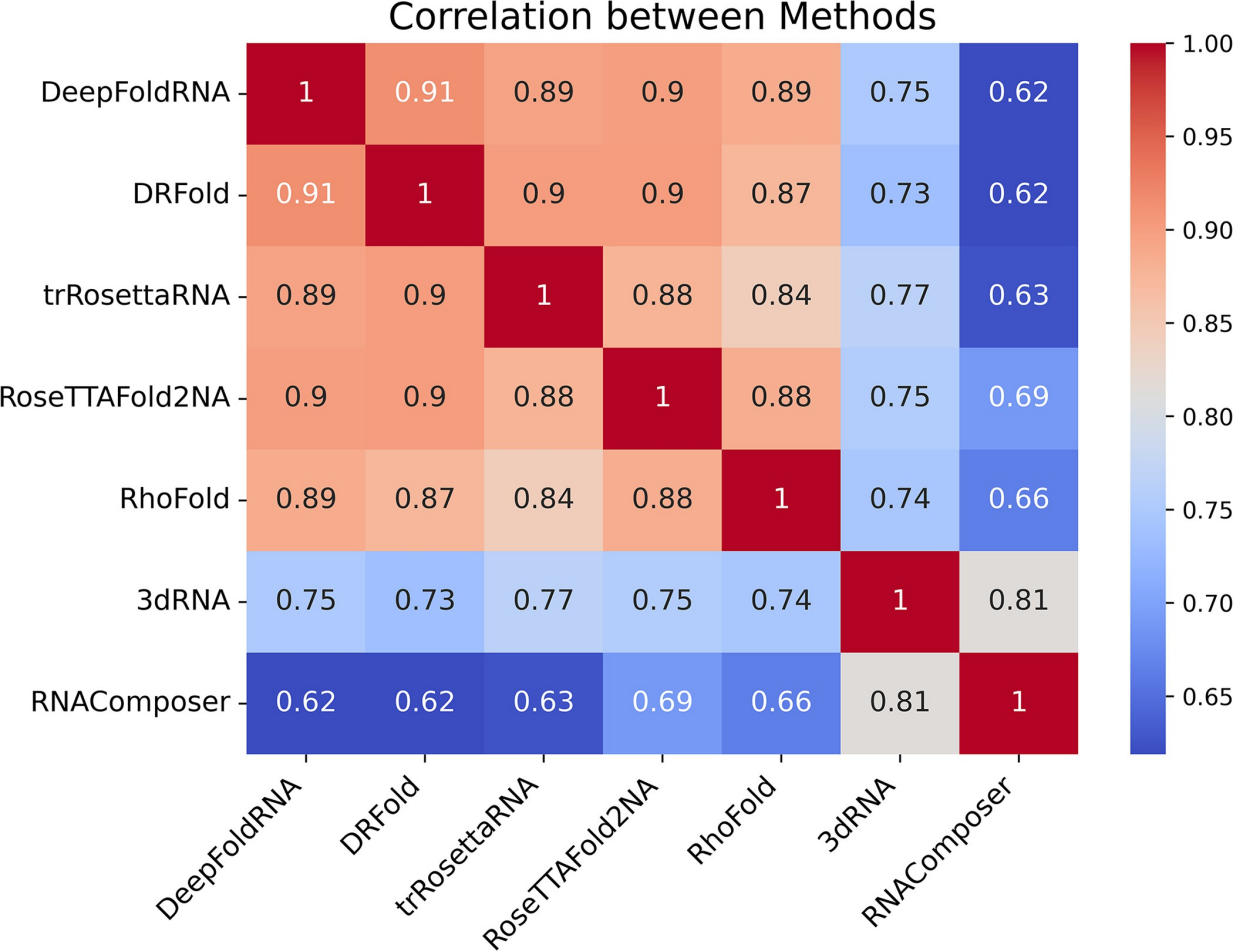
Heatmap showing the correlation between different methods. All the machine-learning-based methods are very similar to each other with DeepFoldRNA and being DRFold having the highest correlation. The fragment-assembly-based methods (3dRNA and RNAComposer) have comparatively lower correlation with ML-based methods and their highest correlation is with each other.

We also analysed the similarity among the prediction methods by calculating the correlations between the methods. We calculated the Pearson correlation coefficients between the RMSD values of the predicted models and visualized the results in a heatmap (Fig 11). Our analysis showed that DeepFoldRNA and DRFold have the highest correlation at 0.91, while all machine learning (ML)-based methods demonstrated strong correlations with one another, averaging around 0.9. In contrast, the FA-based methods showed comparatively lower correlations with the ML methods, with their strongest correlation being at 0.81 between each other.

### Correlation between different metrics

**Fig 12:**
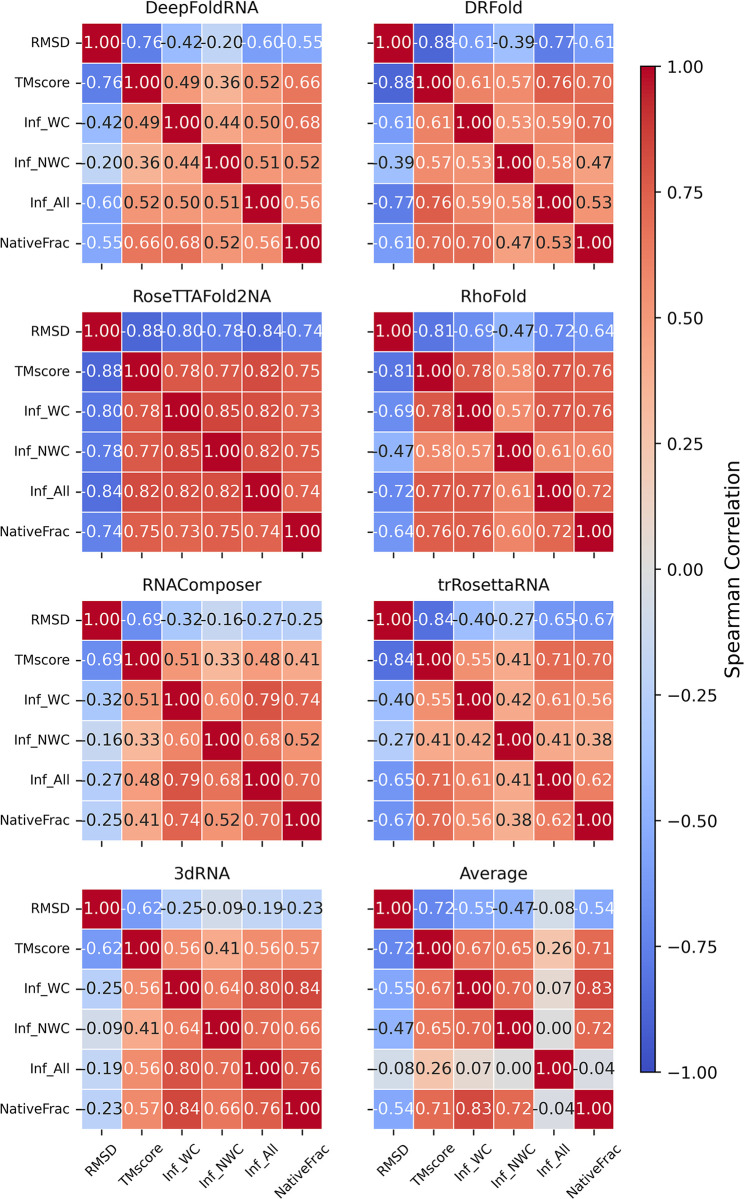
Heatmap showing the correlation of scoring metrics with each other for all the datasets. RMSD has a negative correlation with the remaining metrics as expected (lower the RMSD, better the model). The correlation of the other metrics varies a lot depending on the method with RosettaFold2NA-predicted models having the highest correlation between the scoring metrics. Variation in the similarity of the metrics indicate that different metrics judge different aspects of the model quality, underscoring the importance of using multiple metrics to benchmark the methods.

To assess the similarity between the scoring metrics, we also calculated the correlation between the different metrics that we used for benchmarking the prediction methods. We calculated the spearman correlation of all the scoring metrics (of the predicted model) with each other and plotted a heatmap (Fig 12). We did this analysis for all the seven methods and the average prediction. There was a large variation between the correlations depending on the method, with RosettaFold2NA having the highest agreement between the metrics. This analysis underscores the complexity of RNA structure prediction evaluation and the need for a comprehensive approach with multiple metrics in interpreting the benchmarking results.

### Comparison of ML-based methods with the FA-based methods

**Fig 13:**
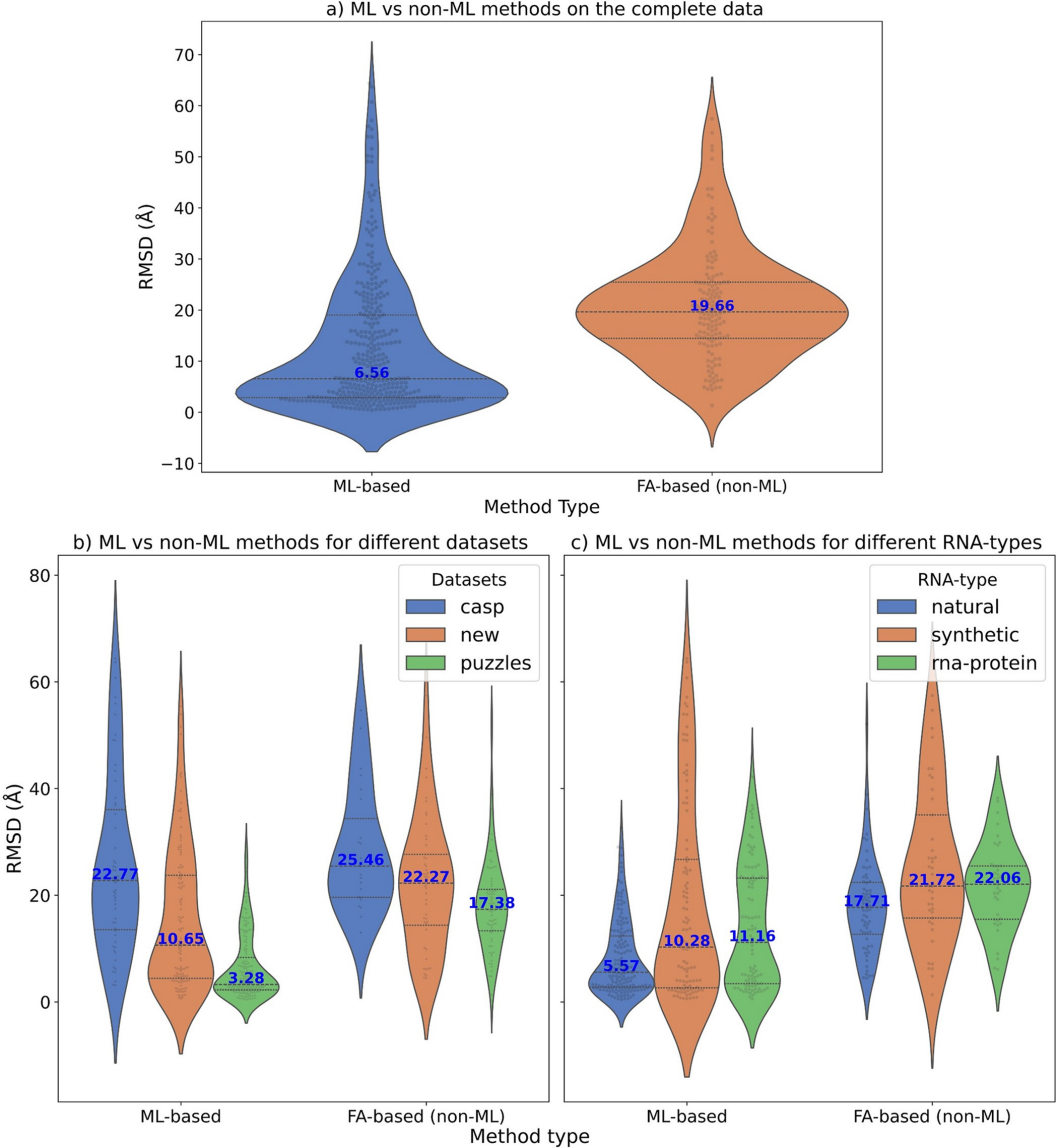
Comparison of ML-based methods to non-ML-based methods. The median values for each violin plot are labelled in blue. The RMSD (in Å) is shown on the y-axis while the x-axis shows the method type (ml or non-ml). **a)** Violin plots showing the distribution of the RMSDs of the predicted models for a comparison between ml (DeepFoldRNA, DRFold, trRosettaRNA, RosettaFold2NA, RhoFold) and non-ml (3dRNA, RNAComposer) methods. The median RMSD of ml methods (6.57 Å) is much lower than non-ml methods (19.66 Å). **b)** Violin plots showing the comparison of ML vs FA-based methods based on different datasets. ML-based methods are clearly better with much lower median RMSD than FA-based methods on the New (10.65 Å vs 22.27 Å) and RNA-puzzles dataset (3.28 Å vs 17.38 Å). ML-based methods are also better than FA-based ones on the CASP15 dataset albeit the difference in median RMSD is not as pronounced (22.77 Å vs 25.46 Å). **c)** Violin plots showing the comparison of ML vs FA-based methods based on different RNA types. ML-based methods are better with much lower median RMSDs for all RNA types. 5.57 Å vs 17.71 Å for natural, 10.28 Å vs 21.72 Å for synthetic and 11.16 Å vs 22.06 Å for RNA-protein complex targets.

We compared the performance of the ML-based methods together against the non-ML or FA-based methods to get an idea about how much better the ML methods are comparatively. The median RMSD of all models predicted by ML-based methods was much lower than the ones predicted by FA-based methods (Fig 13A). We also compared the performance differences based on the datasets (Fig 13B) and RNA type (Fig 13C) and in all cases the ML-based methods were superior to the FA-based methods.

### Effect of different datasets on the prediction accuracy of the methods

**Fig 14:**
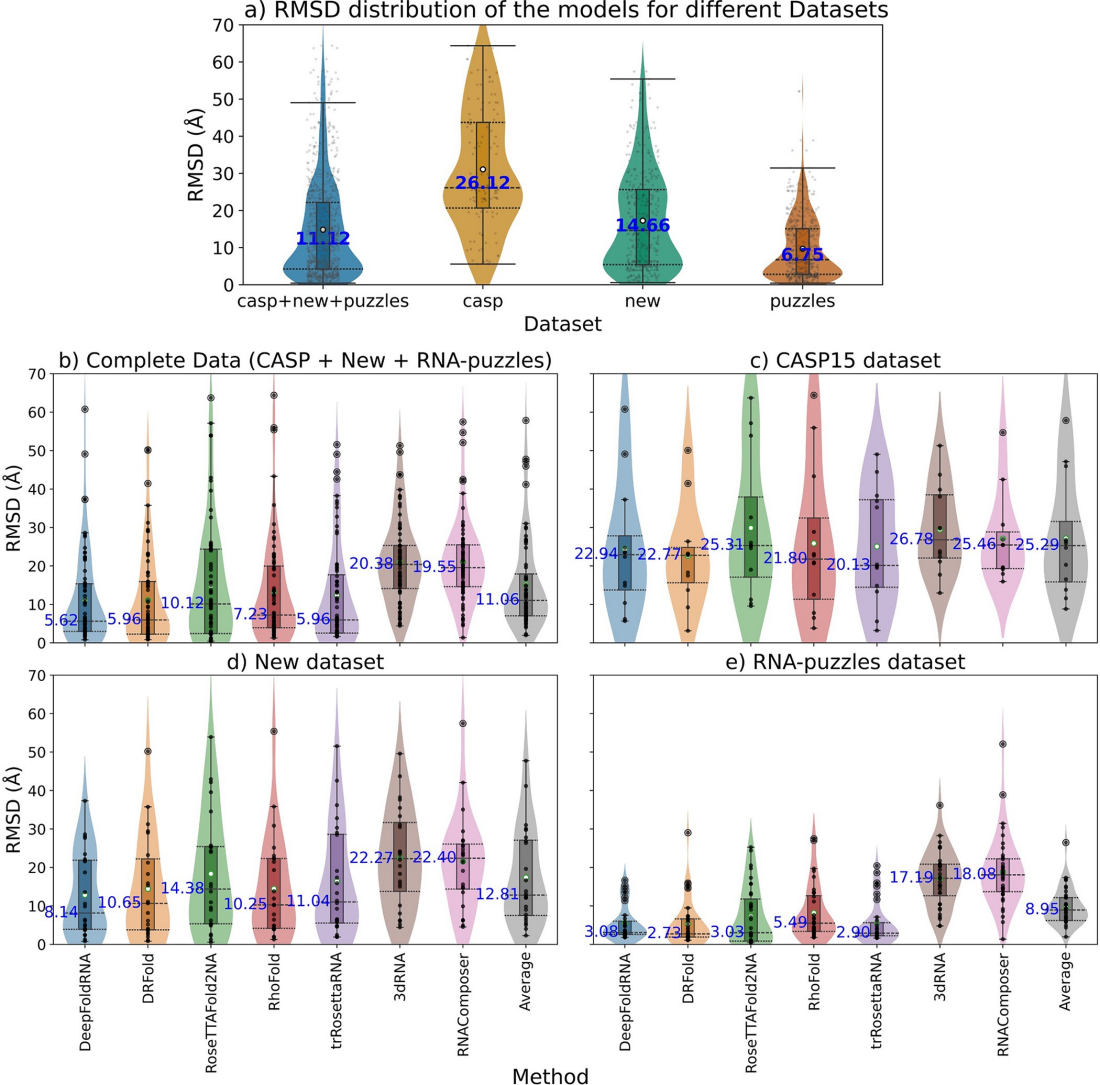
Box and violin plots showing the comparison of the methods based on different datasets. The median values are labelled with blue text and the whiskers denote the interquartile range **a)** The datasets are on the X-axis and the RMSD is on the Y-axis. We pooled all the models from different methods datasets together and only compared the RMSD of the models based on their datasets. The median RMSD for the CASP dataset was the highest (26.12 Å), New dataset was in the middle (14.66 Å) and RNA-puzzles had the lowest median RMSD (6.75 Å). **b)** This plot shows the same comparison, but we look at each method separately. The Average plot (in grey) is the average of the models predicted by all the methods for a particular target. Generally, CASP15 dataset has the highest median RMSD for all methods, New dataset was in the middle and RNA-puzzles dataset has the lowest median RMSD for all the methods. The reason for RNA-puzzles being the easiest is because 35/36 targets are X-ray crystallographic structures and many of the targets were published before 2020, thus they might be included in the training sets of the ML-based methods. CASP dataset is the hardest because most of the targets are synthetic and Cryo-EM structures. The new dataset provides the most realistic performance estimates as it is a well-balanced dataset (comprising all kind of RNAs with representation from both X-ray crystallographic and Cryo-EM structures) and none of its targets are present in the training sets of the ML methods. DRFold has a median RMSD of 2.73 on RNA-puzzles dataset possibly because it has already seen most of the targets in the RNA-puzzles dataset while training thus giving an overinflated performance.

The performance of the methods varies depending on the dataset they are benchmarked on. We compared the accuracy of the models (based on RMSD) for all models depending on the dataset and CASP15 was the hardest, New dataset was in the middle and RNA-Puzzles was the easiest (Fig 14A). We also looked at the performance of each method separately based on the dataset being benchmarked on and we saw similar results (Figs 14B and S5 Fig).

### Effect of RNA type on the prediction performance

**Fig 15:**
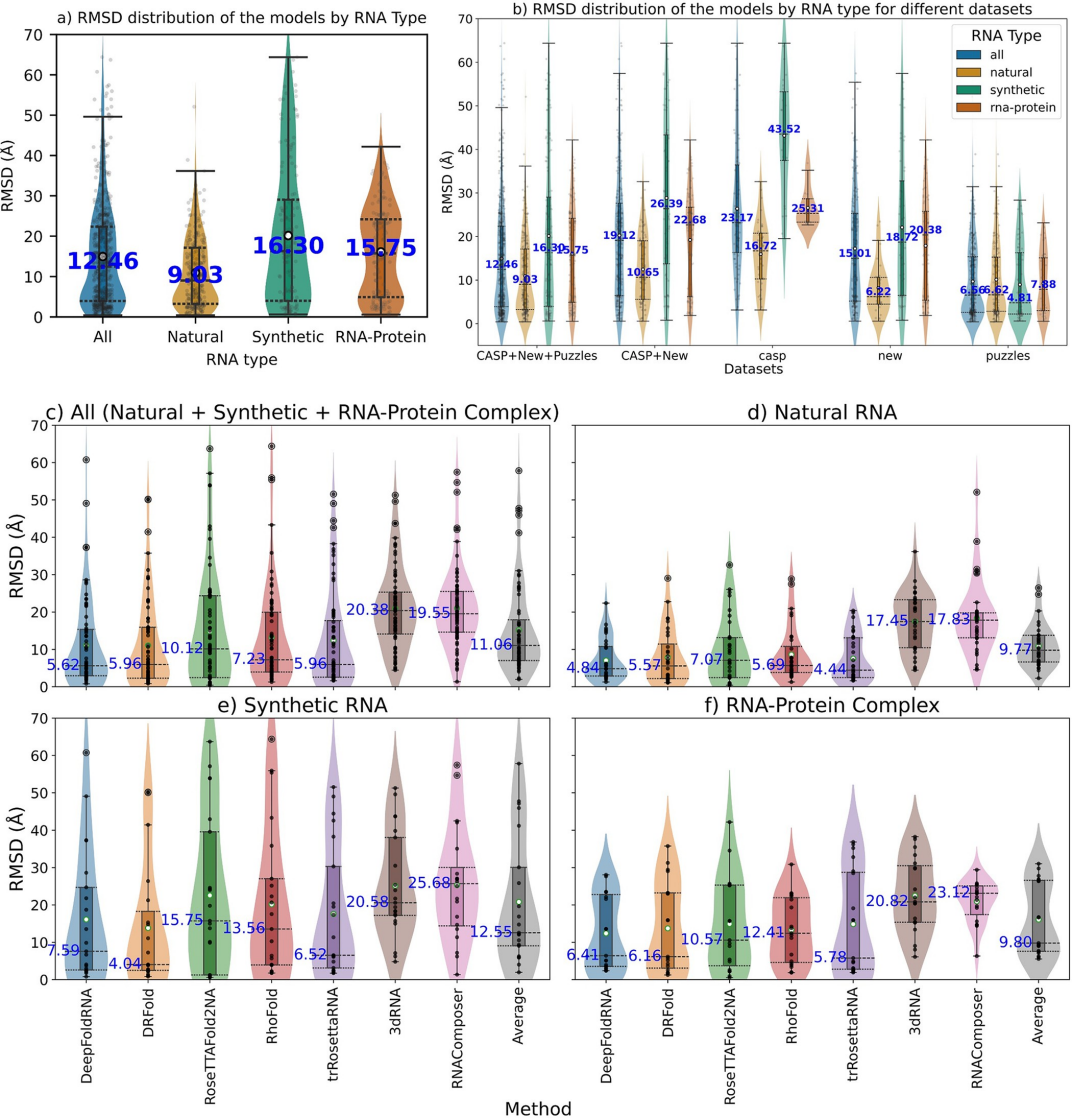
Comparing the prediction performance based on RNA type. **a)** Violin plots showing the RMSDs of the models based on the RNA type. Natural targets have the lowest median RMSD (9.03 Å), RNA-protein have the second best (15.75 Å) and synthetic have the highest (16.30 Å). **b)** Performance difference for different RNA types based on different datasets. For CASP15 dataset, natural have the lowest, RNA-protein have the middle and the synthetic targets have the highest median RMSD. For the new dataset, natural have the lowest (6.22 Å), while RNA-protein and synthetic have similar median RMSDs with synthetic being slightly lower than RNA-protein (18.72 Å for synthetic and 20.38 for RNA-protein). Interestingly for the RNA-puzzles dataset the lowest median RMSD is for the synthetic targets (4.81 Å), while natural RNAs have slightly higher (6.62 Å) and the RNA-protein ones have the highest (7.88 Å). This discrepancy for this dataset is because many of the targets in this dataset are published pre-2020 so they might be present in the training sets of ML-methods thus resulting in an inflated performance (the difficulty of being a synthetic target doesn’t matter because ML-model has already learnt the structure). **c)** Performance comparison of all the methods separately for different RNA types. For all methods (except DRFold) the natural targets are the easiest to predict, with RNA-protein being more difficult and synthetic being the hardest based on the median RMSD scores.

In CASP15, most deep-learning based methods performed poorly on the synthetic targets in CASP15, and also on the RNA-protein complexes none of the methods were accurate. Therefore, we divided all the RNAs into three categories similar to the CASP15-RNA competition: Natural, Synthetic and RNA-protein complex and then compared the prediction performance according to RNA type. The median RMSD of all the models for natural targets was much lower than synthetic and RNA-protein complexes (Fig 15A). We again looked at the same comparison but segregated the models based on data type. We observed that on the CASP dataset, natural targets has the lowest median RMSD, RNA-protein targets were in the middle and synthetic targets had the highest median RMSD, which agrees with the conclusions from CASP15 assessors [56]. For New dataset, natural has the lowest, while synthetic and RNA-protein complex had similar median RMSD (slightly higher for RNA-protein). Interestingly, on the RNA-Puzzles this trend wasn’t observed (Fig 15B) as the synthetic targets actually had lower median RMSD than natural and RNA-protein targets possibly because of the known problem with the RNA-Puzzles dataset (old targets that are part of the training set of ML methods). We also looked at the performance dependence of the methods on the RNA type for all methods separately and found that for all methods (except DRFold) the natural targets are the easiest to predict, with RNA-protein being more difficult and synthetic being the hardest (Figs 15C and S6 Fig).

### Dependency on the length of the RNA

**Fig 16:**
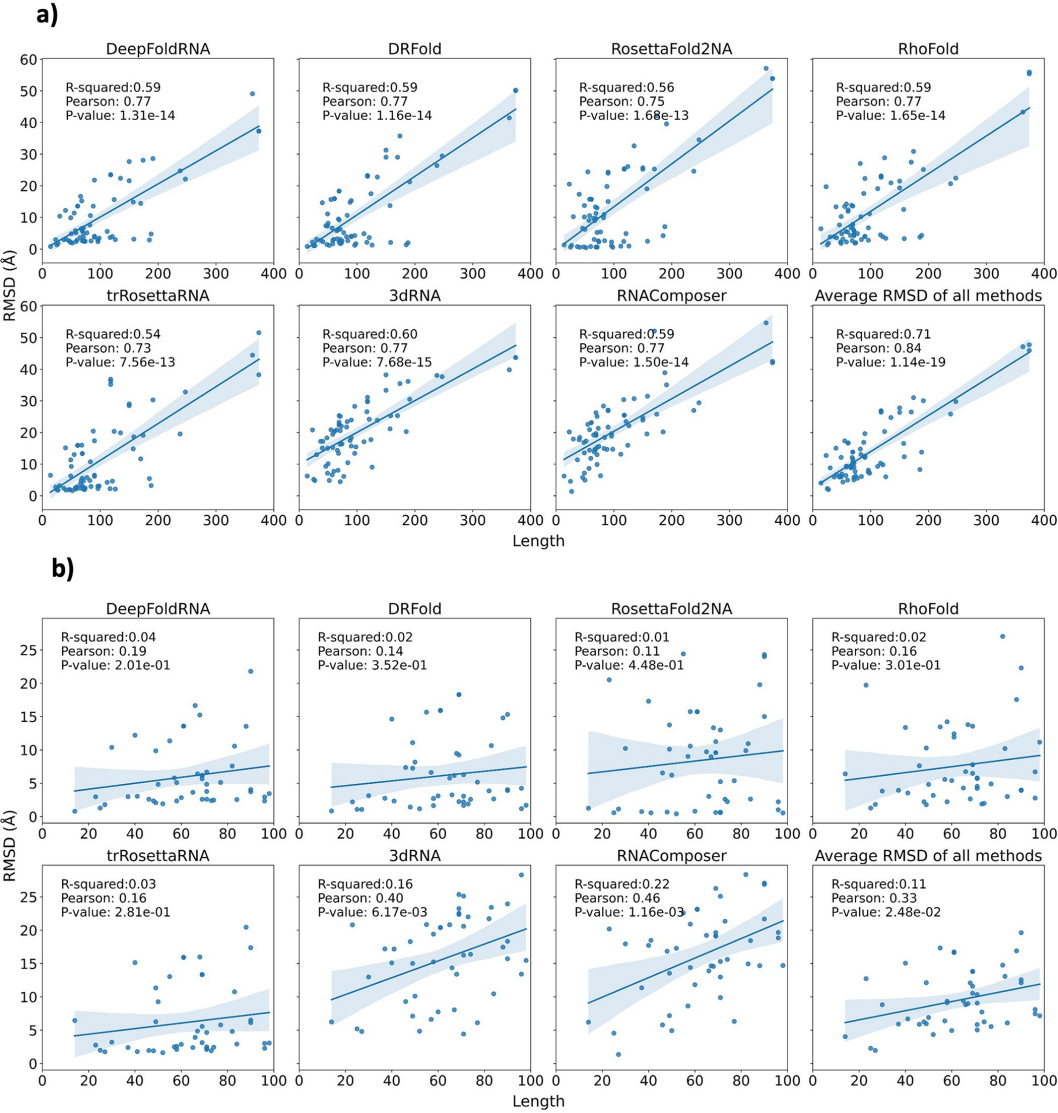
Correlation between the length of the target RNA and the RMSD. **a)** The correlation between the length of the target RNA and the RMSD of the predicted model for all the methods. In this plot RNAs of any length are considered. We observe a positive correlation between the length and the RMSD of the models for all the methods suggesting that longer the RNA, higher the RMSD of the predicted model and hence lower the quality of the predicted model. This shows that predicting the 3D structure of longer RNAs tends to be more challenging than that of shorter RNAs. **b)** The correlation between the length of the target RNAs and the RMSD of the predicted model for all the methods. In this plot, only RNAs with length < 100 are considered. We observe that the clear positive correlation that we observed in (a) for all models is only present for FA-based methods (3dRNA and RNAComposer) and is also much weaker than the first case. The correlation for ML-based methods is not there anymore. This suggests that on increasing length of the target RNA (up to 100 nucleotides) there isn’t much effect on the quality of the predicted model.

**Fig 17:**
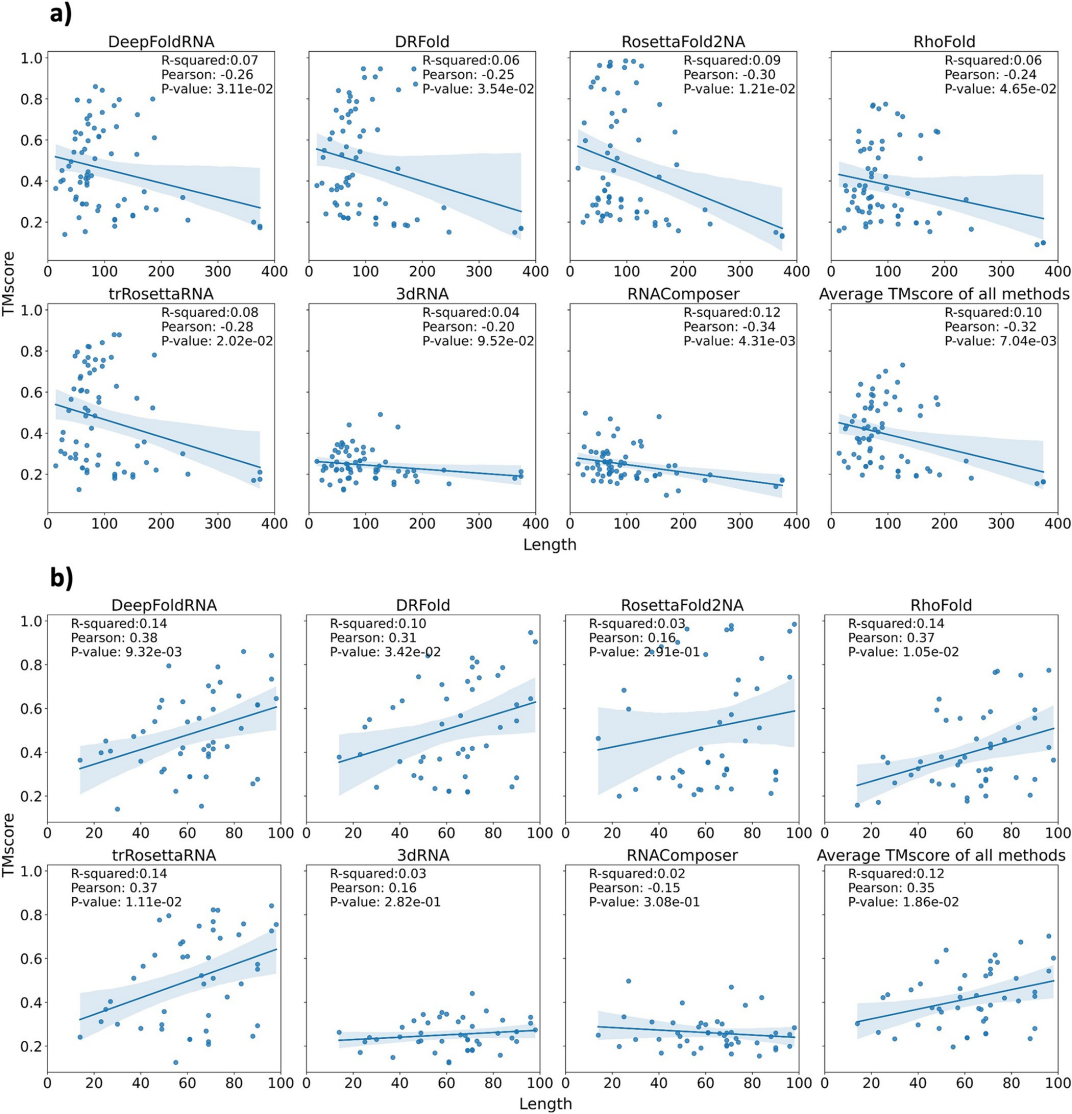
Correlation between the length of the target RNA and the TMScore. **a)** The correlation between the length of the target RNA and the TMscore of the predicted model for all the methods. In this plot RNAs of any length are considered. We observe a negative correlation between the length and the TMscore of the models for all the methods suggesting that longer the RNA, lower the TMscore of the predicted model and hence lower the quality of the predicted model. This could possibly be because most ML-methods are trained on RNAs shorter than 200 nucleotides. **b)** The correlation between the length of the target RNAs and the TMscore of the predicted model for all the methods, in which only RNAs with length < 100 are considered. Contrary to what we expected, the weak negative correlation that we observed in (a) for all models is now a weak positive correlation for most methods. This suggests that on increasing the length of the target RNA (up to 100 nucleotides) the TMscore of the predicted model and hence the quality of the model also increases, which is unexpected, but has been previously reported by the RhoFold paper as well.

We checked the correlation of the length of the target RNA with the RMSD of the predicted models for all the methods (Fig 16A) and as expected, we observed a positive correlation (as length of the target RNA increases, RMSD of the predicted model also increases). For longer RNAs, the RMSD of the predicted models was generally higher than that of the shorter RNAs. However, when we only looked at the correlation for shorter RNAS (RNAs with length < 100), the correlation was much weaker (Fig 16B). On checking the correlation of RNAs of length > 100 with RMSD, we again observed a strong positive correlation (S7 Fig). We observed a negative correlation between TMscore and the length of the RNA (as length increases, TMscore decreases) (Fig 17A), which also agrees with previous literature. In the previously published RhoFold manuscript, a positive correlation between TMscore and the length of the RNA (up to a certain length) has been observed. Therefore, we looked at the correlation between TMscore and length for RNAs of length less than 100 and RNAs of length more than 100 separately. We indeed saw a positive correlation between TMscore and RNA length for RNAs of length less than 100 (Fig 17B). The correlation was again negative for RNA length > 100 (S8 Fig). This indicates that both shorter and longer RNAs are difficult to predict because of certain confounding factors.

### Effect of the quality of MSA on the RNA

**Fig 18:**
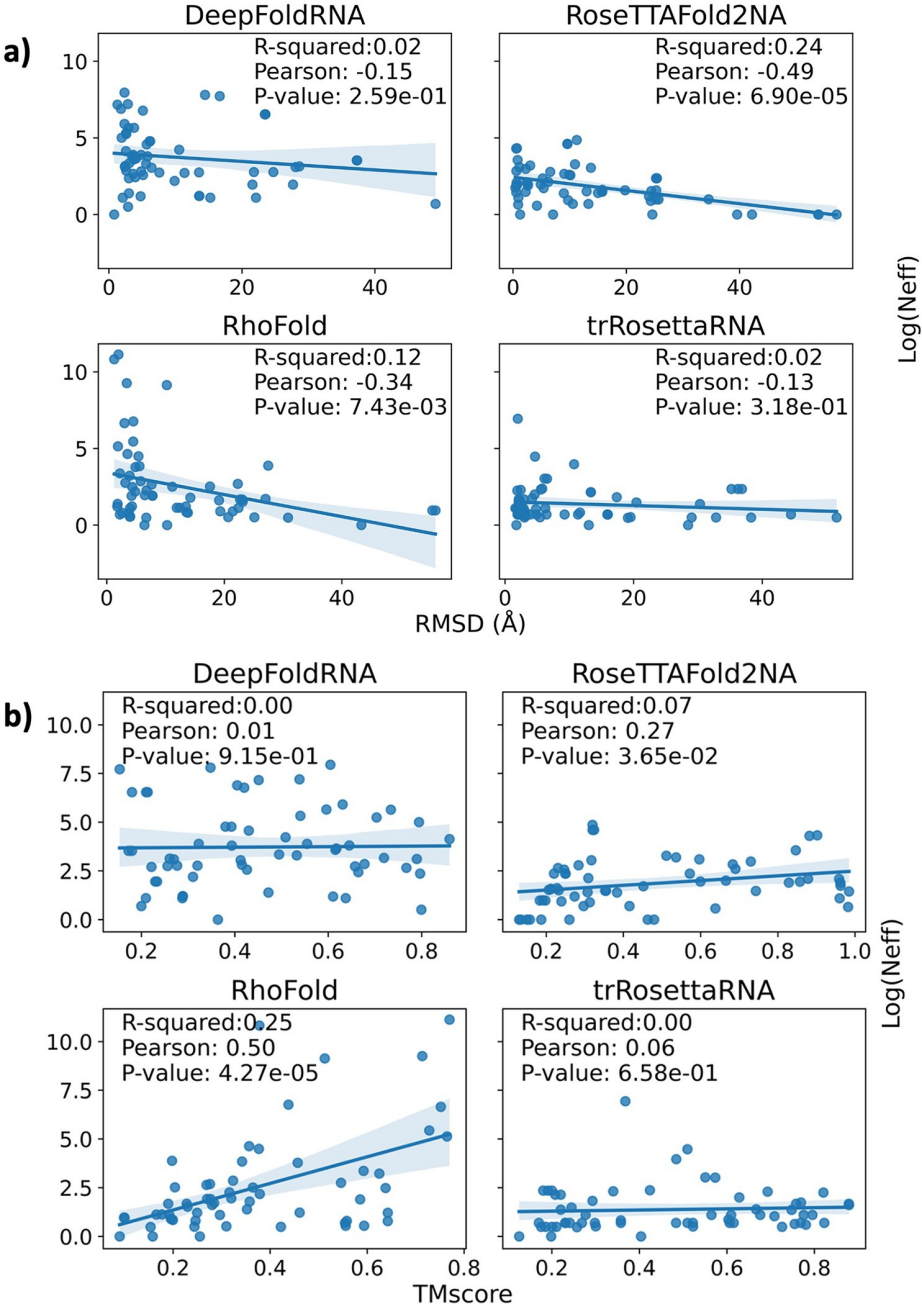
Correlation between RMSD/TMscore and the MSA depth. Scatter plots showing the correlation between RMSD/TMscore and the MSA depth (Log(N_eff_)) for the four ML-based methods (DeepFoldRNA, RosettaFold2NA, trRosettaRNA, RhoFold) that take MSA as input. DRfold was excluded as it doesn’t take MSA as input. As the methods used by the tools to create the MSA differ, we created separate scatter plots for each of the methods. DeepFoldRNA uses rMSA, RoseTTAFold2NA uses rMSA-lite, trRosettaRNA uses a mix of rMSA and Infernal, while RhoFold only relies on blastN to create the MSA. **a)** A negative correlation is observed for RoseTTAFold2NA and RhoFold which indicates that models of the targets with higher MSA depth have better quality (lower RMSDs). **b)** Scatter plots for the four methods between TMscore and the MSA depth (Log(Neff)). A positive correlation is observed for RoseTTAFold2NA and RhoFold which indicates that models of the targets with higher MSA depth have higher TMscore.

The depth of the MSA used as input to deep-learning-based structure prediction methods has previously been suggested to affect the quality of the predicted models. Recently, Lee et al. found that expanding the MSA database boosts ColabFold’s CASP15 performance [89]. Therefore, we investigated the effect of MSA quality on the RMSD of the predicted models. The differences in the MSA tools (rMSA for DeepFoldRNA, rMSA-lite for RosettaFold2NA, rMSA + Infernal for trRosettaRNA, blastN for RhoFold) and the RNA databases that are used to create the MSA can cause a lot of variance in the quality of the MSA for the same target sequence [90]. We used the Log(N_eff_) measure to quantify the quality of the MSA depth. The N_eff_ metric is calculated from the hmmbuild command in the HMMAlign [91] tool and is a measure of the number of effective homologous sequences in the alignment. We don’t observe a correlation between the RMSD and the MSA depth for DeepFoldRNA and trRosettaRNA, but a negative correlation is observed for RoseTTAFold2NA and RhoFold, which indicates that higher-depth MSA is more informative and results in better quality prediction for these tools (Fig 18A). If we look at the correlation between the TMscore of the targets and their MSA depth, we again observe a positive correlation for RoseTTAFold2NA and RhoFold, while that is not the case for DeepFoldRNA and trRosettaRNA (Fig 18B).

### Effect of secondary structure on the prediction performance

**Fig 19:**
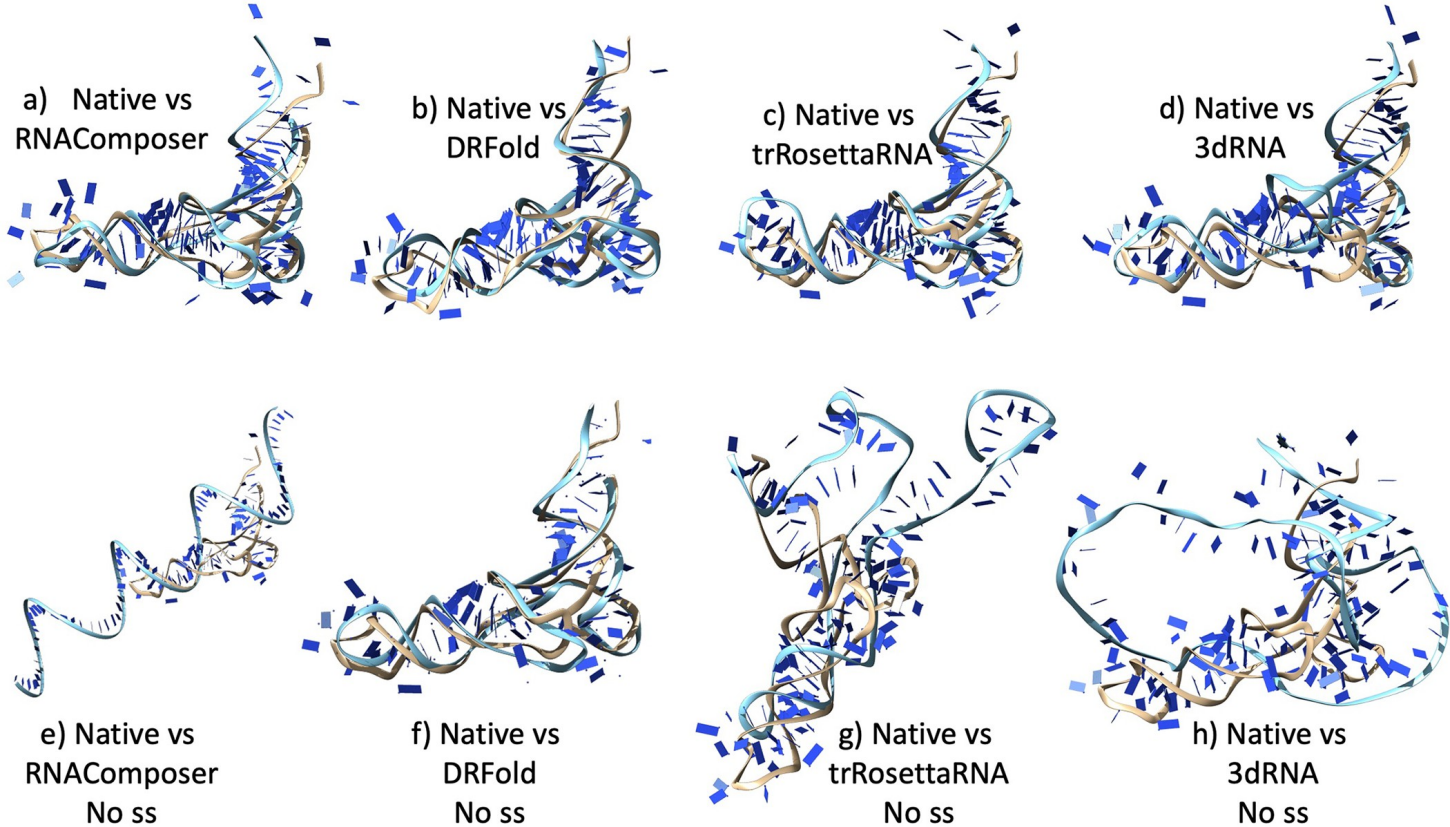
Effect of ss on 8FZR model predictions. Superimposition of the native structure and predicted models of the target 8FZR showing the comparison between the quality of the predicted models by various tools when secondary structure (ss) is provided as input (predicted by the respective prediction method of each tool; SPOT-RNA for trRosettaRNA, RNAfold for RNAComposer and 3dRNA, and PETfold + RNAfold for DRFold) and when no secondary structure input is given. The native structure is shown in the light grey colour and the predicted models are in cyan. The first row of structures i.e. Fig 19a, 19b, 19c, and 19d show the superimposition from the case when secondary structure is provided as input and the bottom row i.e. Fig 19e, 19f, 19g, and h show the superimposition from the scenario when no secondary structure is provided as input. When ss is not provided as input, we can clearly see (in Fig 19e, 19f, 19g, 19h) that the quality of the predicted models by all the methods (except DRFold) is far worse than the models from the first row (in Fig 19a, 19b, 19c, 19d). This indicates that although replacing the predicted ss by extracted ss from native PDBs as input to these tools didn’t improve the quality of the final predicted model substantially, removing the ss as input altogether severely affects the quality of the final predicted model. Therefore, ss still plays a very important role in the accurate determination of the 3D RNA structure. The only method that wasn’t greatly affected by exclusion of ss as input was DRFold (possibly because it’s able to predict the nucleotide pairing and the associated restraints somewhat accurately even in the absence of ss because of how it’s trained the geometrical potentials it uses to fold the RNA; recall that it doesn’t take an MSA as input). The RMSD between the native and modelled structures are as follows: **a)** 5.70 Å for RNAComposer model **b)** 4.42 Å for DRFold model **c)** 5.78 Å for trRosettaRNA model **d)** 5.52 Å for 3dRNA model **e)** 59.46 Å for RNAComposer model without ss as input **f)** 4.63 Å for DRFold without ss as input **g)** 25.73 Å for trRosettaRNA model without ss as input **h)** 24.35 Å for 3dRNA model without ss as input.

The secondary structure (ss) of the RNA plays an important role in the tertiary folding of the RNA molecule, therefore, it’s imperative to use the correct ss as input to the DL-model [92]. However, we also saw in the INF plots that the percentage of correctly predicted WC-pairs was on average already around 80% for the predicted models, which means that for most of the targets the correct ss information is already provided as input (predicted by respective third-party ss prediction tool or the internal Neural network architecture). Nevertheless, we investigated how using the correct ss (extracted from the native PDB files) as input will improve the model prediction. We selected 14 targets that have their complete native structure available (no residues missing) and extracted the ss from their PDB files using the RNAPDBee 2.0 tool [93]. We observe that with the native ss as input the fragment-assembly-based methods (RNAComposer and 3dRNA) show a large improvement in the quality of the models, while for ML-based methods, we don’t see a huge difference (slight increase for DRFold and slight decrease in trRosettaRNA) in the final model quality (Table 9). The probable reason for this could be that the ML-based methods are actually trained on ss input from their respective ss prediction methods so ss extracted from native PDBs might not offer much improvement in the quality of the predicted model and native ss as input rather creates restraints that these tools are not able to handle, thus resulting in models with higher RMSDs. We also tried removing the input ss information to the methods completely for one of the targets (8FZR) and predicted the final model without any input ss information. The quality of the final predicted model dropped substantially for all methods (except DRFold) on removing the ss information altogether (Fig 19).

**Table 9:** RMSD comparison (in Å) of models for 13 targets predicted by methods that take secondary structure (ss) as input. The methods compared are RNAcomposer (rnac), 3dRNA (3dRNA), DRFold (drfold) and trRosettaRNA (trr). The columns without ‘_ss’ suffix indicate the predicted models where the input ss is the default one predicted by the respective associated ss prediction method for each tool (RNAfold for RNAComposer and 3RNA; SPOT-RNA for trRosettaRNA; PETfold + RNAfold for DRFold). The columns with ‘_ss’ suffix are the ones where the input ss is the one extracted from the native PDB file using the RNAPDBee tool. We observe that for both the fragment-assembly-based methods i.e. RNAComposer and 3dRNA, the average RMSD for predicted models is much lower with the native ss compared to the default ones (22.64 Å vs 13.72 Å for RNAComposer and 24.81 Å vs 20.65 Å for 3dRNA). For the ML-based methods i.e. DRFold and trRosettaRNA, we don’t see much difference between the two scenarios. We actually observe a slight increase in the average RMSD for DRFold (17.27 Å vs 18.19 Å) and a slight reduction in the average RMSD for trRosettaRNA (19.80 Å vs 18.63 Å). The probable reason for this could be that the ML-based methods are actually trained on ss input from their respective ss prediction methods so ss extracted from native PDBs might not offer much improvement in the quality of the predicted model and native ss as input rather creates restraints that these tools are not able to handle, thus resulting in models with higher RMSDs.

Target	rnac	rnac_ss	3drna	3drna_ss	drfold	drfold_ss	trr	trr_ss
**7u0y**	15.45	12.02	8.1	9.26	6.03	7.98	4.18	5.02
**7uri**	31.79	20.84	20.19	20.46	16.09	17.05	15.77	16.72
**7wi9**	16.25	16.09	17.19	16.39	16.4	16	17.19	17.18
**7zj5**	42.49	NA	44.18	38.76	50.2	53.47	51.98	39.23
**8bgu**	23.9	14.32	24.95	10.22	3.57	3.46	4.53	4.37
**8dp3**	21.67	6.14	24.47	16.02	15.31	13	17.65	16.53
**8eyu**	13.6	15.74	15.43	13.76	11.81	11.94	11.36	9.8
**8fcs**	10.24	6.57	3.98	4.86	2.54	2.6	4.21	5.84
**8gxb**	23.53	11.37	20.95	9.37	16.57	15.3	15.91	9.03
**r1107**	19.78	12.51	22.77	22.82	18.52	17.41	13.24	6.67
**r1128**	27.53	18.46	38.68	29.3	26.71	36.1	19.83	25.76
**r1138**	NA	NA	51.33	48.07	NA	NA	44.44	45.15
**r1190**	25.5	16.82	30.32	29.21	23.47	23.98	37.09	40.93
**Average**	**22.64**	**13.72**	**24.81**	**20.65**	**17.27**	**18.19**	**19.8**	**18.63**

### Results on the RNA3DB dataset

**Fig 20:**
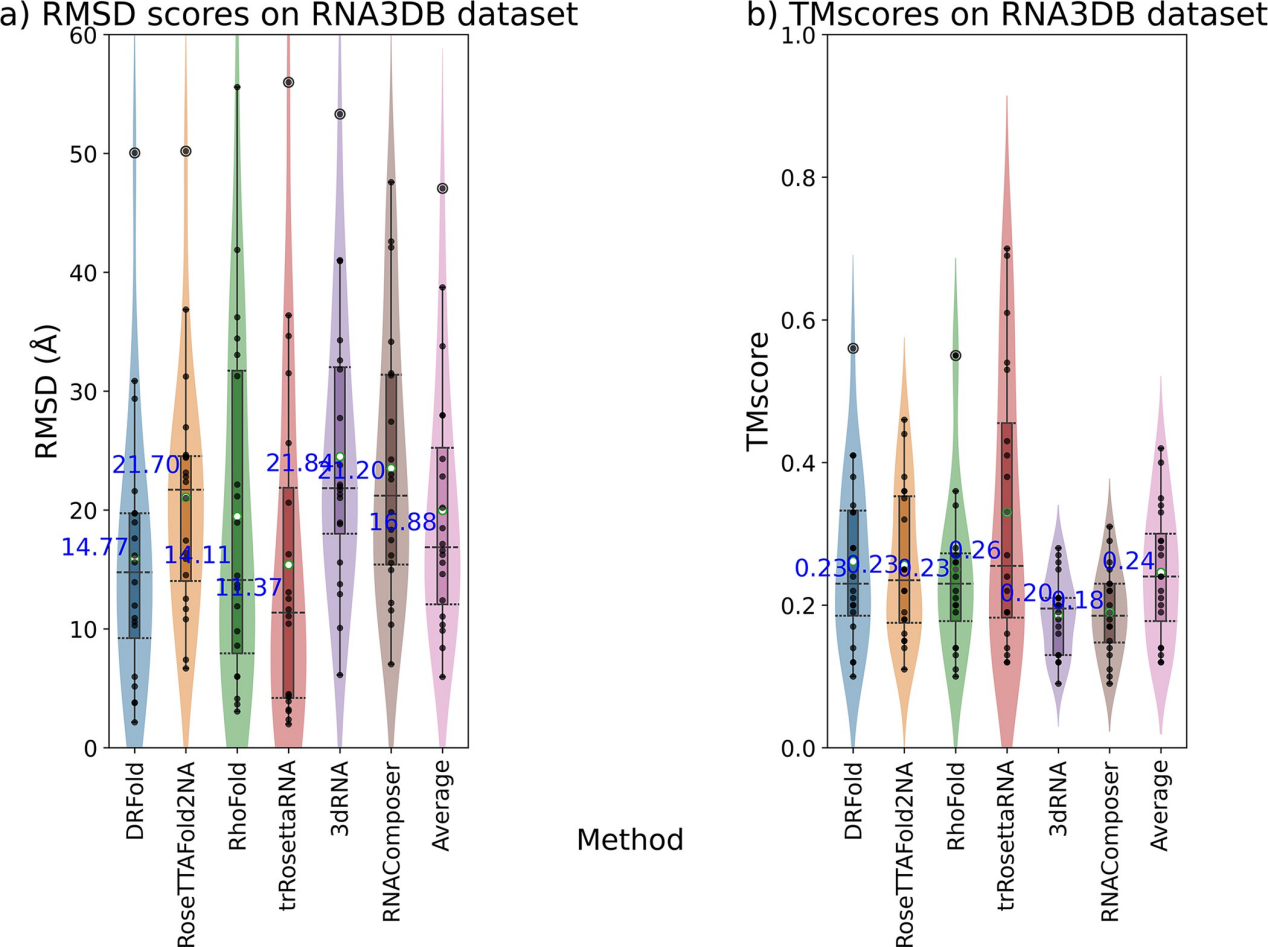
Box and violin plots showing the benchmarking results on the RNA3DB dataset. The methods are on the X-axis and the metrics are on the Y-axis. The Average plot (in violet) is the average of the models predicted by all the methods for a particular target. The median values are labelled with blue text and the whiskers denote the interquartile range **a)** RMSD distribution of the predicted models by various methods. trRosettaRNA has the lowest median RMSD (11.37 Å). **b)** TMscore distribution for the various methods. trRosettaRNA the highest median TMscore of 0.26.

We observed that three ML-based methods (DRFold, RhoFold, and trRosettaRNA) have a lower mean/median RMSD compared to the average and FA-based methods (Table 10 and Fig 20A). RosettaFold2NA-predicted models had worse RMSD than the average and its performance was almost similar to the FA-based methods. The performance difference between ML-based methods and FA-based wasn’t as substantial as observed in other datasets, and overall even our best method (trRosettaRNA) had a mean RMSD of 15.38 Å. As this dataset is composed of mostly orphan RNA and is quite challenging for the prediction methods, the prediction performance of all the methods (including ML-based methods) barring some targets is poor. This is similar to what’s been observed for Alphafold, that as the availability of multiple sequence alignments (MSA) decreases, particularly for orphan proteins, the performance of AlphaFold diminishes significantly [94]. We also observe that the method that solely rely on MSA i.e. RosettaFold2NA has a much poorer performance compared to the methods that either use ss-prediction methods (trRosettaRNA, DRFold) or language models (RhoFold). We saw similar results (ML-based methods being slightly better than average/FA-based methods), when we looked at the TMscore of the predicted models with the best method having a median TMscore of only 0.26 (Fig 20B). Overall, trRosettaRNA was the best method (in terms of RMSD and TMscore) on this dataset, followed by DRFold and RhoFold. We also looked at other metrics of the predicted models and most of them indicated the substantially lower prediction performance of the benchmarked-methods on this dataset (S9 Fig).

**Table 10:** Comparison of RMSD values (in Å) of the predicted models to the native structure for targets in the RNA3DB dataset. trRosettaRNA has the lowest average RMSD (15.38 Å) on this dataset followed by the DRFold method (15.91 Å). RMSD for the fragment-assembly-based methods (3DRNA and RNAComposer) is higher than that of the deep-learning-based methods, but the difference is comparatively much smaller than on the other datasets. RosettaFold2NA has almost similar performance to the FA-based methods as the MSA depth of orphan RNA’s is poor.

^#^Target	drfold	rf	rhofold	trr	rnac	3drna
**8ane_r**	19.71	36.87	36.22	55.99	42.6	41.03
**8d1v_j**	13.93	16.39	34.44	34.65	34.16	34.29
**8d29_r**	5.98	6.68	6.01	3.91	7.04	6.13
**8d4a_b**	3.77	15.9	5.98	1.98	15.56	18.82
**8do6_j**	11.97	26.96	13.73	13.09	23.01	22.15
**8e0f_d**	2.14	11.68	3.06	2.37	27.44	27.75
**8ewg_b**	21.59	22.81	18.95	20.62	16.19	21.02
**8f0n_b**	10.59	10.81	8.59	3.22	12.2	13.77
**8ff5_m**	50.05	50.2	55.58	25.64	47.58	53.31
**8ffy_c**	10.3	24.5	9.81	4.29	19.82	18.94
**8gkh_w**	29.37	24.65	21.15	31.51	17.47	21.7
**8gxb_b**	17.62	17.43	14.48	4.45	24.26	21.34
**8h7q_d**	18.97	22.38	41.88	36.38	42.09	40.95
**8hb8_a**	15.6	24.41	13.43	3.09	22.58	23.79
**8hud_b**	16.17	21.02	3.66	16.28	18.34	21.98
**8ibw_d**	19.75	14.53	22.14	12.53	11.56	12.92
**8j1q_e**	10.91	12.53	11.9	10.44	10.36	10.09
**8sqj_p**	30.86	31.24	31.27	11.08	31.51	31.83
**8sqj_t**	5.16	23.16	33.06	11.65	31.34	32.6
**8upt_a**	3.82	7.41	4.12	4.52	14.96	15.59
**Average**	**15.91**	**21.08**	**19.473**	**15.38**	**23.50**	**24.50**

^#:^ We weren’t able to benchmark DeepFoldRNA on this dataset due to technical reasons.

drfold: DRFold, rf: RosettaFold2NA, rhofold: RhoFold, rnac: RNAComposer, trr: trRosettaRNA, 3drna: 3dRNA

## Discussion and conclusion

In this study, we conducted a comprehensive benchmarking of various RNA structure prediction methods across diverse datasets, each presenting varying levels of difficulty. While our primary focus was on deep-learning-based approaches, we also incorporated fragment-assembly methods to check the comparative effectiveness of machine learning (ML) versus traditional techniques. We evaluated seven 3D RNA structure prediction methods on three datasets, encompassing a total of 66 target RNAs. The benchmarked methods comprised five ML-based (deep-learning) approaches and two non-ML-based (fragment-assembly) methods. Performance assessment took into account multiple factors such as RNA length, MSA, dataset characteristics, and RNA types. Generally, ML methods exhibited significantly superior performance compared to their non-ML counterparts. The effectiveness of the methods varied based on the benchmarking dataset, with the CASP15 dataset posing the greatest challenge as this dataset included numerous targets that were either synthetic or RNA-protein complexes. In contrast, the RNA-Puzzles dataset proved to be the easiest, possibly because it contains many targets published before 2020 which might have been part of the ML methods’ training datasets. Notably, the RNA-Puzzles dataset predominantly comprised X-ray crystallography structures, while CASP15 mainly consisted of Cryo-EM structures. Given that X-ray crystallography structures generally have better resolution and accuracy, the ML methods, trained predominantly on crystallographic structures, exhibited better accuracy when modelling X-ray targets. The newly compiled dataset has a good balance of Cryo-EM and X-Ray structures where none of the targets were seen by the ML-methods previously and therefore this dataset provides the most realistic benchmarking set for evaluating the methods in a blind prediction scenario. On this dataset, DeepFoldRNA had the lowest median RMSD. We would like to also point out that in CASP 15 competition, RNAComposer was one of the top performing methods, but this was because of carefully selected secondary structure elements and human experts. As previously stated, this benchmarking focuses on fully automatic predictions (considering only the default ss prediction methods and the default input parameters for each method). We also looked at the performance of the different methods stratified by the difficulty of the target RNAs and, DeepFoldRNA (for medium and hard targets) and DRFold (for easy targets) had the best performance.

The performance was also affected by the type of RNAs and the length of the RNA. Natural RNAs were the easiest targets with best quality models, while RNA-protein complex models had medium accuracy, and the synthetic targets were the hardest. We also observed that generally the quality of the predicted models (based on RMSD) gets lower as the sequence length of the target RNA increases, although, the correlation is quite weak when looking at RNAs of length less than 100 nucleotides. Similar trend was seen when we looked at the correlation of length vs TMscore with longer RNAs having lower TMscore. However, we also observed a positive correlation between length and TMscore when only considering RNAs of sequence length less than 100. Generally, when trying to predict the structure of very long RNAs, the model quality is less accurate because the prediction tool is not able to predict long-range interactions, pseudoknots and the associated base-pairing. This is because getting a good quality MSA (non-sparse and high depth) for longer sequences is difficult as very few homologous sequence matches are found. However, when the sequences are too short then the deep-learning-models don’t have enough information to work with, diminishing the predicted model quality. This indicates that there is an optimum range for the length of RNA for the best prediction quality.

We also observed a slight correlation between the MSA and model quality for RosettaFold2NA and RhoFold methods, while there wasn’t any correlation observed for the other ML-methods (DeepFoldRNA and trRosettaRNA), which suggested that methods that only take MSA as input have worse performance than methods that take MSA + secondary structure (ss) as input. In simpler terms, the negative impact of using a lower quality MSA as input is more pronounced in the predictions generated by RoseTTAFold2NA and RhoFold compared to DeeFoldRNA and trRosettaRNA. One plausible explanation for this could be that DeepFoldRNA and trRosettaRNA utilize existing tools for predicting the secondary structure (PETFold for DeepFoldRNA and SPOT-RNA for trRosettaRNA) [95,96] and then use both the MSA and the secondary structure as input to their transformer network, while in the case of RoseTTAFold2NA and RhoFold the sole input to their neural network architecture is the MSA. Thus, even if the MSA is sparse, DeepFoldRNA and trRosettaRNA can compensate by leveraging the input ss information to predict the correct 1D and 2D geometries, whereas RoseTTAFold2NA and RhoFold have to rely solely on the MSA, which might lead to comparatively higher inaccuracies in predicting the base-pairing information and the associated restraints. These inaccuracies propagate downstream into the final prediction resulting in models with lower TMscore. In our benchmarking on the RNA3DB dataset, we also observed the same thing that due to this being an orphan RNA dataset, the depth of the MSA’s was lower which lead to poorer models being predicted by the ML-methods on this dataset. RosettaFold2NA had a much poorer performance on the RNA3DB dataset (similar performance to the FA-based methods) because of its reliance on MSA, while trRosettaRNA and DRfold have a comparatively better performance as they can rely on the input ss structure in the absence of good quality MSA (non-sparse).

We also checked the dependence of the model quality on the input ss. Although, using ss extracted from native PDBs instead of predicted ss as input didn’t improve the predicted model quality for the ML-based methods by much, removing the input ss altogether reduced the model quality substantially for most methods (except DRFold). This underscores the importance of incorporating ss information in RNA structure prediction, even if the predicted ss may not always enhance model quality substantially.

Altogether, the DL-based methods had the best predicted models with DeepFoldRNA being the best followed by DRFold as the close second. The DL-based methods had the best predicted models for most targets except for the synthetic targets in CASP15 dataset. The FA-based methods lagged behind for most targets and therefore for fully automatic predictions we recommend to rely on the ML-based methods for predicting 3D RNA structures moving forward. When working with synthetic targets, all of the methods have a poor performance and none of them can predict a reliable 3D structure. If modelling a target RNA with known information about complex ss elements, or a synthetic RNA, or an orphan RNA (no homologues with poor MSA) we will recommend to still rely on FA-based methods with human expert input as they outperform automated DL-methods as shown in CASP15. For future method development, we recommend to develop methods that use both MSA and secondary structure as input. Increasing the MSA depth by using metagenomic datasets has shown improvements in case of AlphaFold and we recommend to follow suit for RNA structure prediction as well. Deeper MSAs for RNAs can be constructed using the new RNA database published by Chen at al. [97]. To improve the input ss we recommend to use a ss predicted by ensemble methods, especially the latest ML-based methods. Even the best median INF-nwc score among all the methods was less than 50%, which suggested that none of the methods are able to predict the non-Watson-crick pairs accurately. Therefore, including ss prediction methods that can predict non-canonical base pairs, pseudoknots and other complex RNS ss elements more accurately should also be taken into account for future method development. The biggest challenge in RNA structure prediction still remains the lack of experimental 3D structures, therefore we hope that as more RNA experimental structures are solved and the size of the RNA PDB database increases with better representation of RNAs from previously uncharacterized Rfam families, the ML-based methods will improve as well.

## Supporting information

S1 Fig CASP 15 results. Scatterplot showing the performance of the predicted models based on various metrics for the targets in the CASP15 dataset.
(TIF)

S2 Fig New dataset results. Scatterplot showing the performance of the predicted models based on various metrics for the targets in the New dataset.
(TIF)

S3 Fig RNA-puzzles dataset results. Scatterplot showing the performance of the predicted models based on various metrics for the targets in the RNA-puzzles dataset.
(TIF)

S4 Fig Pairwise comparison of different methods based on TMscore. Scatterplot showing the performance comparison of each method against every other method on all the targets based on TMscore. If a point lies on the red-coloured x = y line, it indicates that the TMscore of the predicted model from both the methods is exactly the same i.e. they have similar prediction performance for that target. Points above that line indicate a higher TMscore for the model predicted by the method on the y-axis (i.e. method on the y-axis is better) and points below that line indicate vice-versa. Most of the ML-based methods have a better performance than the average prediction (last row of plots), while the FA-based methods are much worse than the average prediction (Average vs 3dRNA and Average vs RNAComposer plots in the last row). When compared against all other methods using the TMscores of the predicted models, DeepFoldRNA and DRFold are the best methods (DeepFoldRNA is slightly better).
(TIF)

S5 Fig RMSD cut-off plot for all the methods based on different datasets. RMSD cut-off plots for the seven methods based on the datasets they are benchmarked on. At an RMSD cut-off of 10 Å, in CASP15 none of the methods are able to even predict 30% of the targets correctly, while in the New dataset most ML-methods are able to correctly predict about 40% or more targets correctly (DeepFoldRNA predicts almost 50% targets correctly, the Average (in grey) correctly predicts about 38% targets correctly). In the RNA-puzzles dataset, most ML-methods have a correct prediction rate of 60% or higher with some even surpassing 80% (DRFold, DeepFoldRNA and trRosettaRNA). The Average method (in grey) for RNA-puzzles dataset at 10 Å predicts 62% of the targets correctly. This overinflated performance of ML-methods for RNA-puzzles dataset is because they have many of the targets in their training set.
(TIF)

S6 Fig RMSD cut-off plot for all the methods based on different RNA types. RMSD cut-off plots for the seven methods based on the RNA type. At an RMSD cut-off of 10 Å, for Natural RNAs the ML methods are able to predict 65% to 80% of the targets correctly (DeepFoldRNA and RhoFold are able to predict almost 80% of the natural RNA targets correctly), while in the case of Synthetic and RNA-protein complexes the % of correctly predicted targets is much lower (50% for synthetic by the best method and 59% for RNA-protein complex).
(TIF)

S7 Fig Correlation between RMSD and Length of the RNAs for RNAs longer than 100 nucleotides. The correlation between the length of the target RNAs and the RMSD of the predicted model for all the methods. In this plot, only RNAs with length > 100 are considered. We see a positive correlation between RMSD and length indicating that as the RNA length increases the model quality decreases.
(TIF)

S8 Fig Correlation between TMscore and Length of the RNAs for RNAs longer than 100 nucleotides. The correlation between the length of the target RNAs and the TMscore of the predicted model for all the methods. In this plot, only RNAs with length > 100 are considered. We see a negative correlation between RMSD and length indicating that as the RNA length increases the TMscore decreases, thus the model quality also decreases.
(TIF)

S9 Fig Benchmarking results on the RNA3DB dataset. Results on the RNA3DB dataset. **a)** Native contact fraction (ncf) of the predicted models by the various methods. trRosettaRNA has the highest median ncf of 0.43. **b)** Interaction network fidelity (INF) score for the various methods. trRosettaRNA has the highest median INF score (0.70) **c)** INF-wc score (Watson-Crick pairs) for the various methods. trRosettaRNA has the highest median score of 0.63. Most of the methods have a low INF-WC score. **d)** INF-nwc score (non-Watson-Crick pairs) for the various methods. All of the methods have a median INF-nwc score of 0 (or close to 0), indicating that all of them fail to predict any non-canonical interaction pairs in these orphan RNAs.
(TIF)
