# Supplementary figures and images for: Systematic benchmarking of deep-learning methods for tertiary RNA structure prediction
Source: PLoS Comput Biol. 2024 Dec 30;20(12):e1012715. doi: 10.1371/journal.pcbi.1012715 (PMC11723642; doi:10.1371/journal.pcbi.1012715)

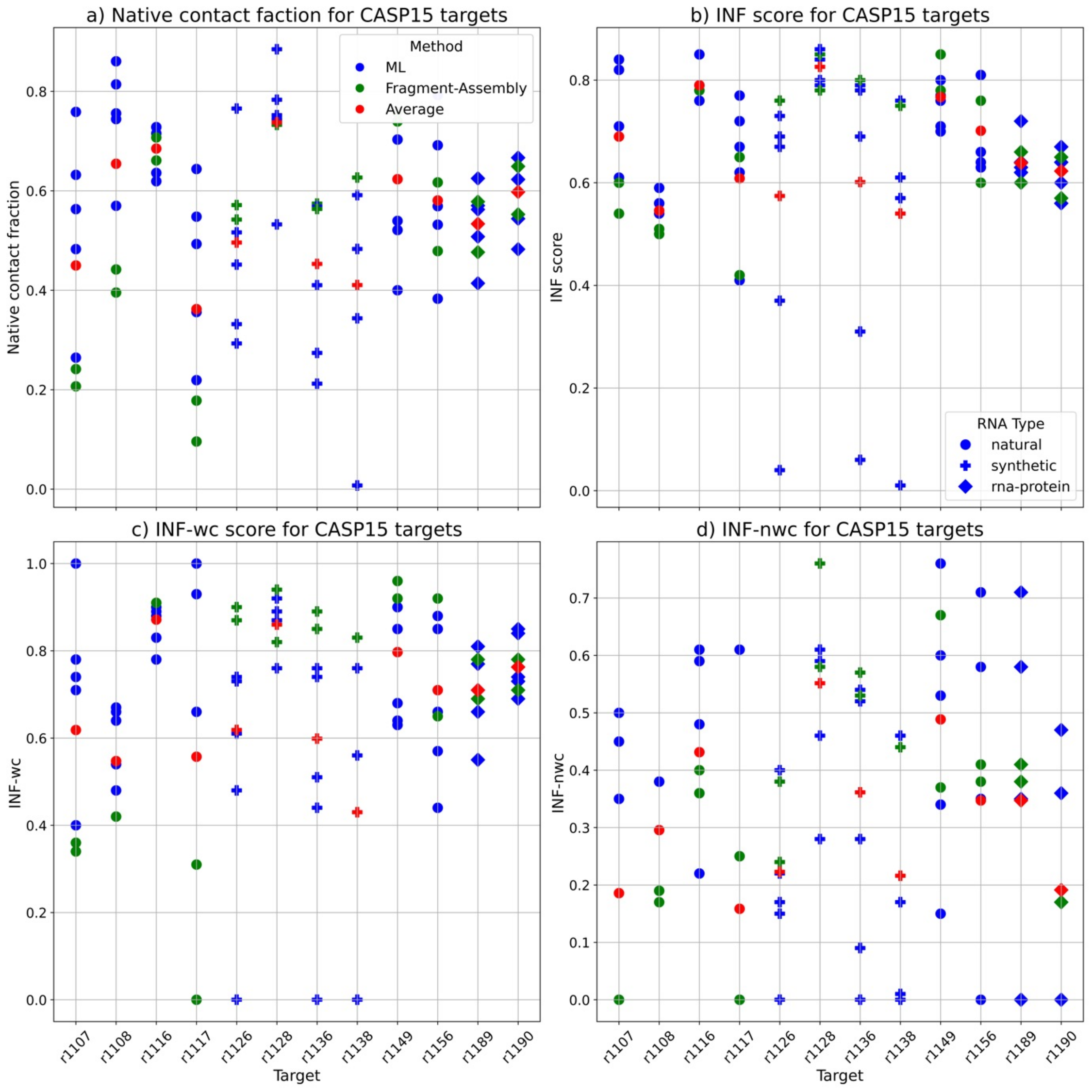

Supplement: S1 Fig — Scatterplot showing the performance of the predicted models based on various metrics for the targets in the CASP15 dataset. (TIF) [file pcbi.1012715.s001.tif]

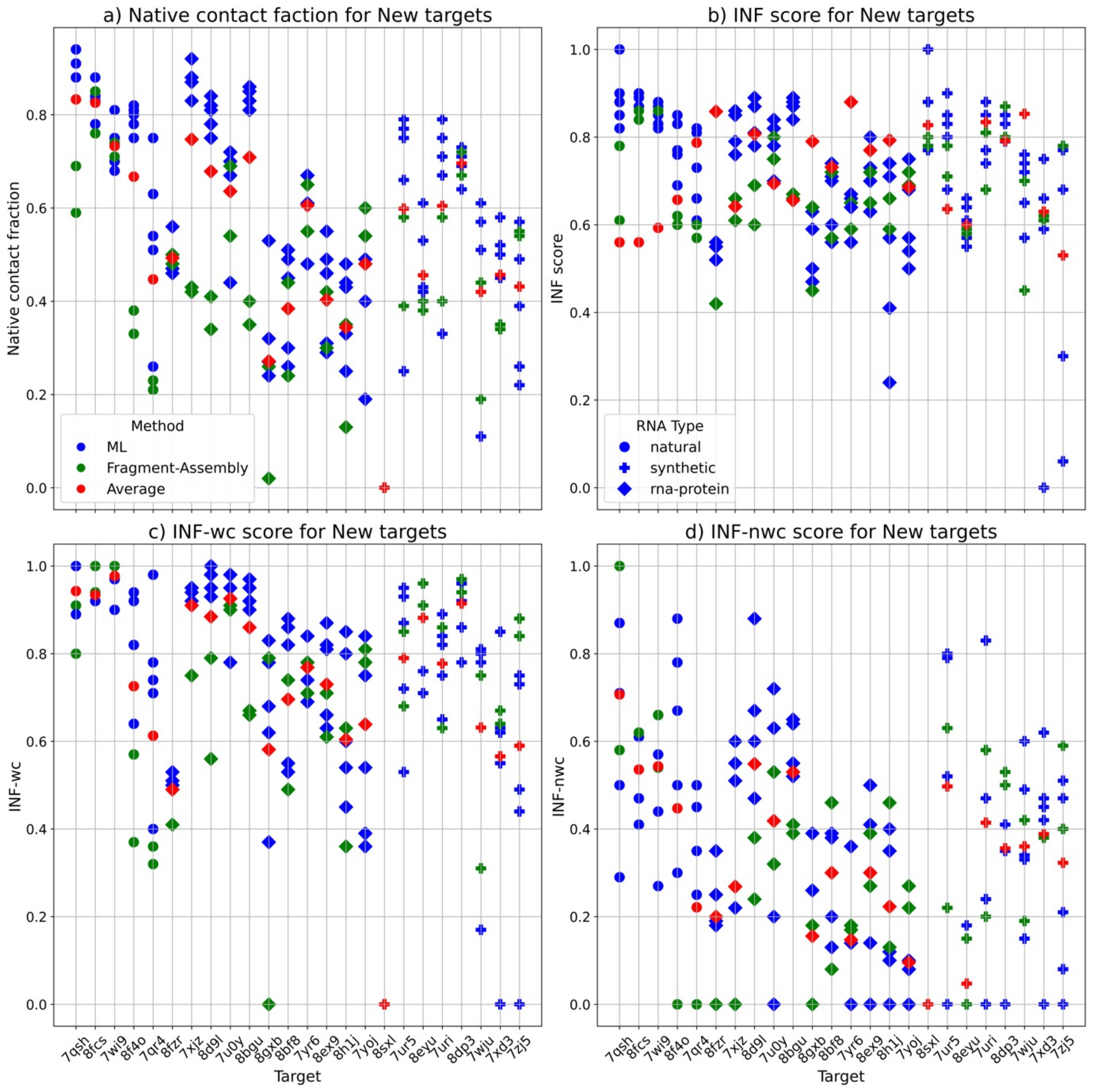

Supplement: S2 Fig — Scatterplot showing the performance of the predicted models based on various metrics for the targets in the New dataset. (TIF) [file pcbi.1012715.s002.tif]

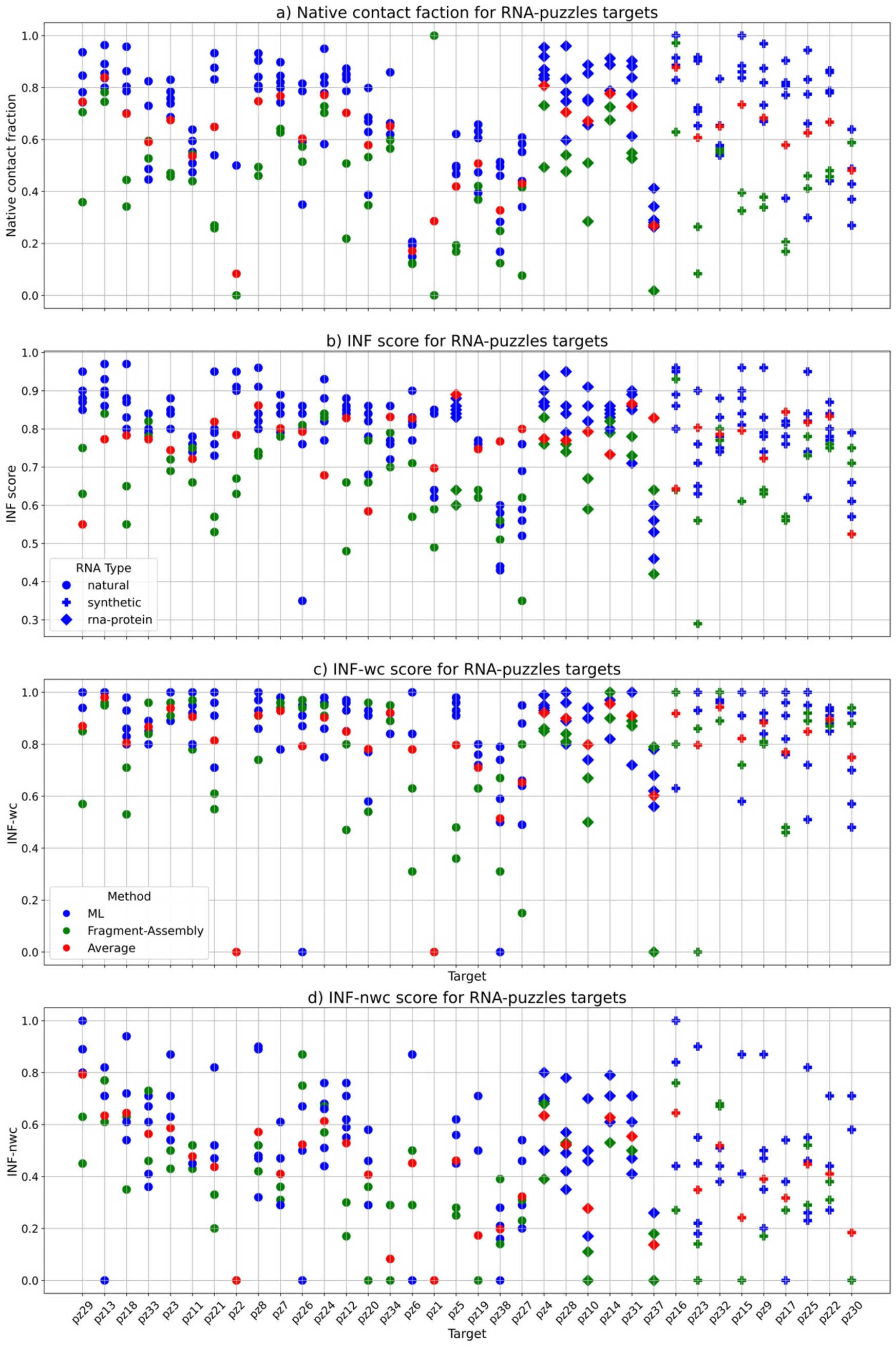

Supplement: S3 Fig — Scatterplot showing the performance of the predicted models based on various metrics for the targets in the RNA-puzzles dataset. (TIF) [file pcbi.1012715.s003.tif]

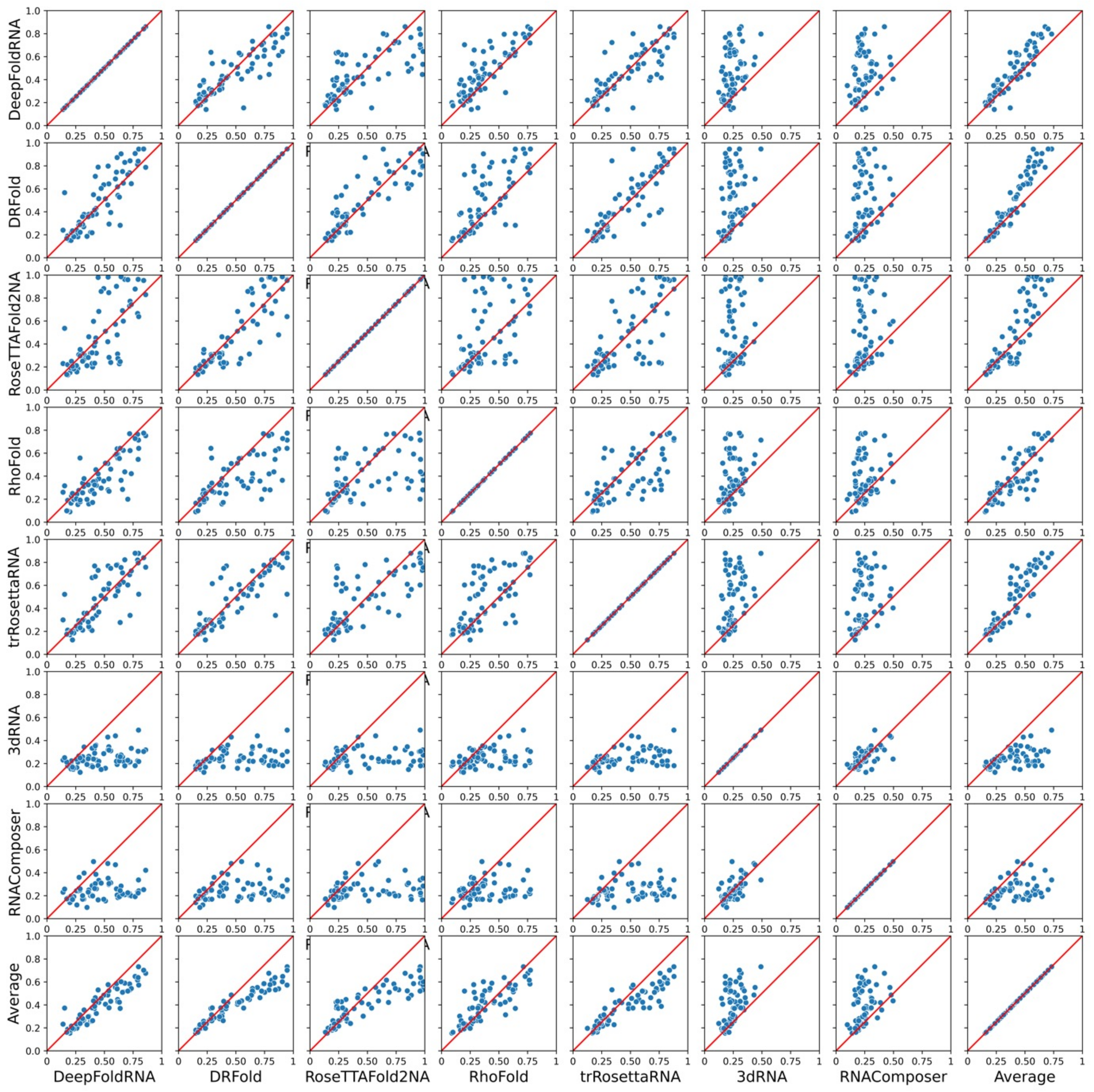

Supplement: S4 Fig — Scatterplot showing the performance comparison of each method against every other method on all the targets based on TMscore. If a point lies on the red-coloured x = y line, it indicates that the TMscore of the predicted model from both the methods is exactly the same i.e. they have similar prediction performance for that target. Points above that line indicate a higher TMscore for the model predicted by the method on the y-axis (i.e. method on the y-axis is better) and points below that line indicate vice-versa. Most of the ML-based methods have a better performance than the average prediction (last row of plots), while the FA-based methods are much worse than the average prediction (Average vs 3dRNA and Average vs RNAComposer plots in the last row). When compared against all other methods using the TMscores of the predicted models, DeepFoldRNA and DRFold are the best methods (DeepFoldRNA is slightly better). (TIF) [file pcbi.1012715.s004.tif]

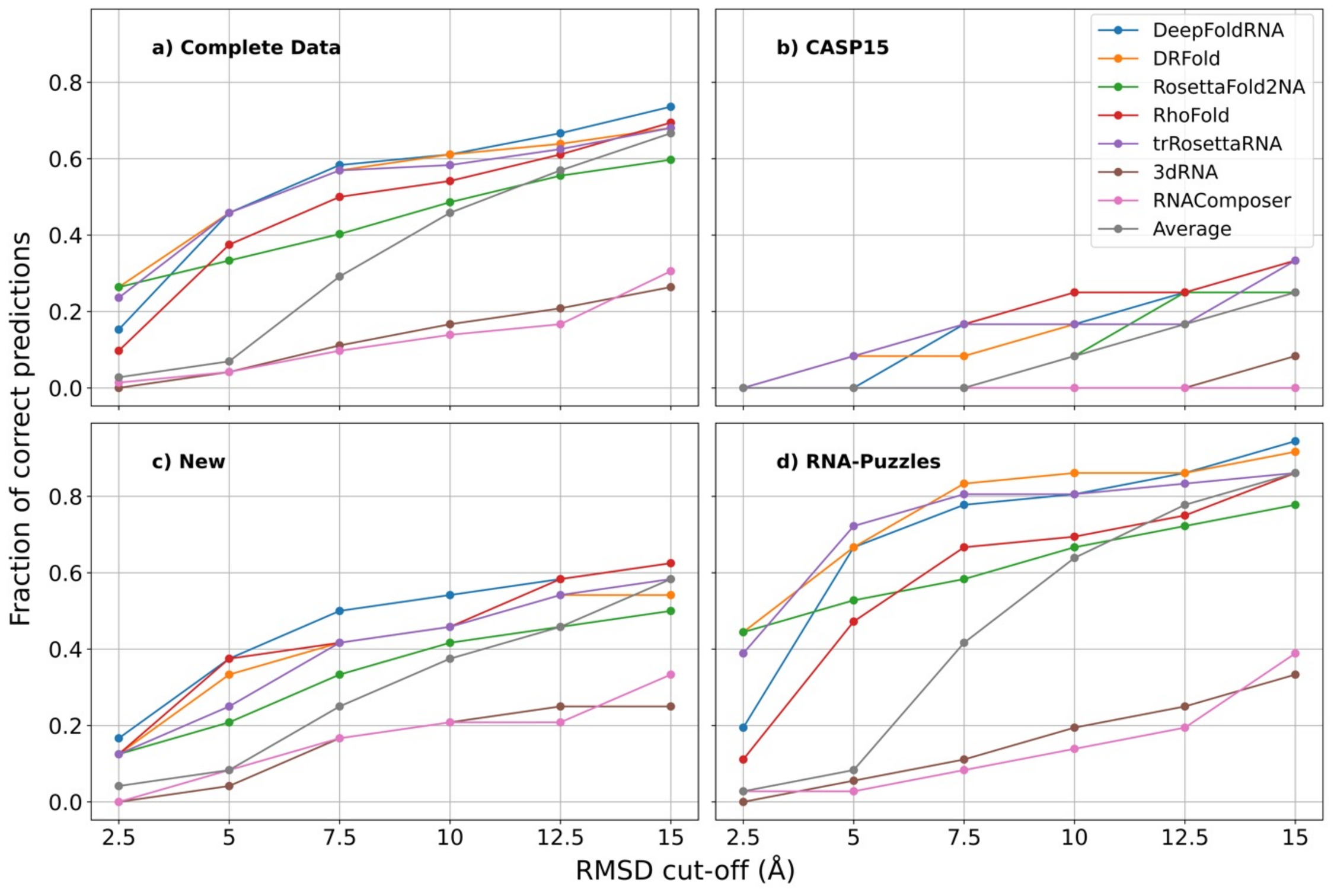

Supplement: S5 Fig — RMSD cut-off plots for the seven methods based on the datasets they are benchmarked on. At an RMSD cut-off of 10 Å, in CASP15 none of the methods are able to even predict 30% of the targets correctly, while in the New dataset most ML-methods are able to correctly predict about 40% or more targets correctly (DeepFoldRNA predicts almost 50% targets correctly, the Average (in grey) correctly predicts about 38% targets correctly). In the RNA-puzzles dataset, most ML-methods have a correct prediction rate of 60% or higher with some even surpassing 80% (DRFold, DeepFoldRNA and trRosettaRNA). The Average method (in grey) for RNA-puzzles dataset at 10 Å predicts 62% of the targets correctly. This overinflated performance of ML-methods for RNA-puzzles dataset is because they have many of the targets in their training set. (TIF) [file pcbi.1012715.s005.tif]

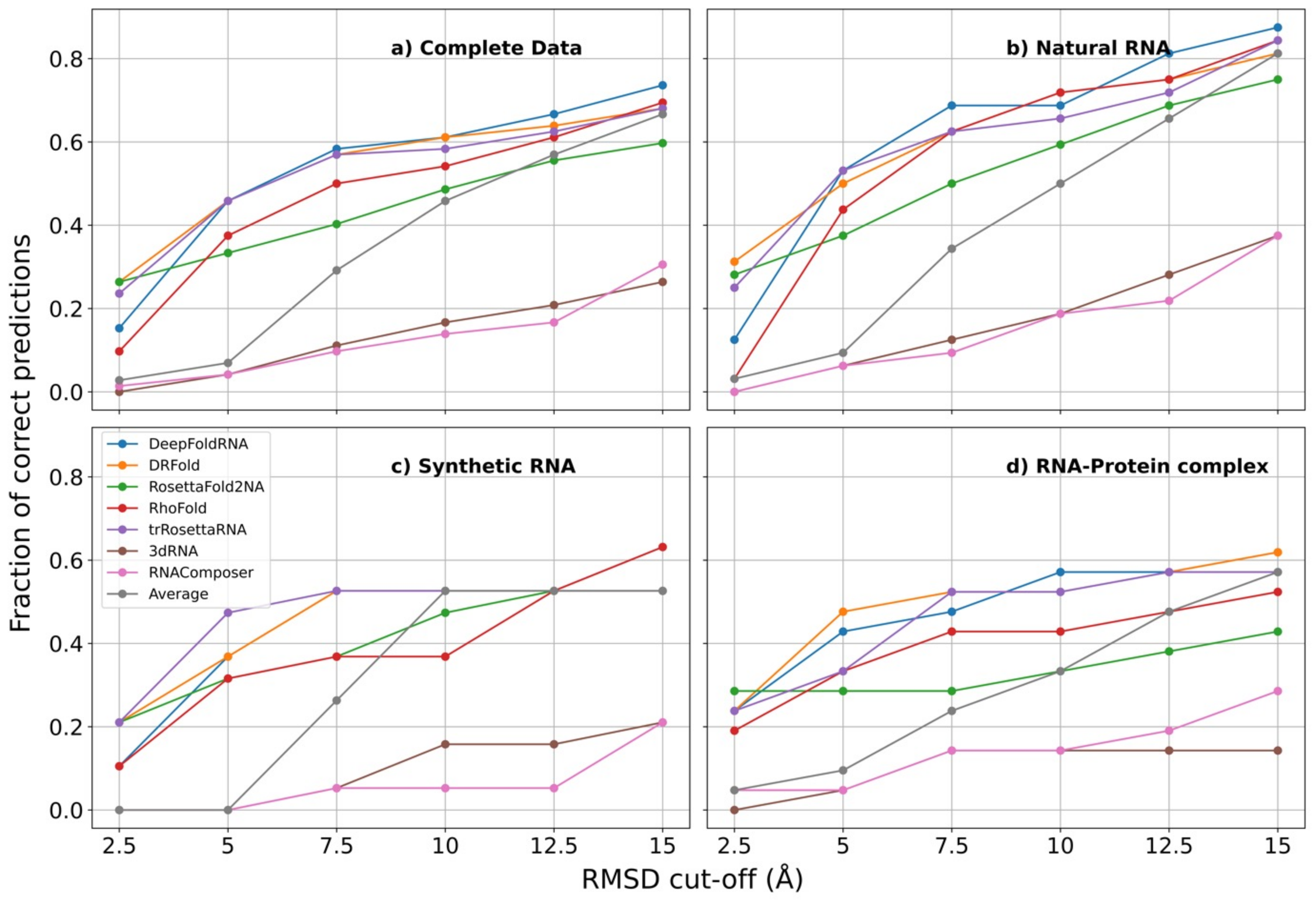

Supplement: S6 Fig — RMSD cut-off plots for the seven methods based on the RNA type. At an RMSD cut-off of 10 Å, for Natural RNAs the ML methods are able to predict 65% to 80% of the targets correctly (DeepFoldRNA and RhoFold are able to predict almost 80% of the natural RNA targets correctly), while in the case of Synthetic and RNA-protein complexes the % of correctly predicted targets is much lower (50% for synthetic by the best method and 59% for RNA-protein complex). (TIF) [file pcbi.1012715.s006.tif]

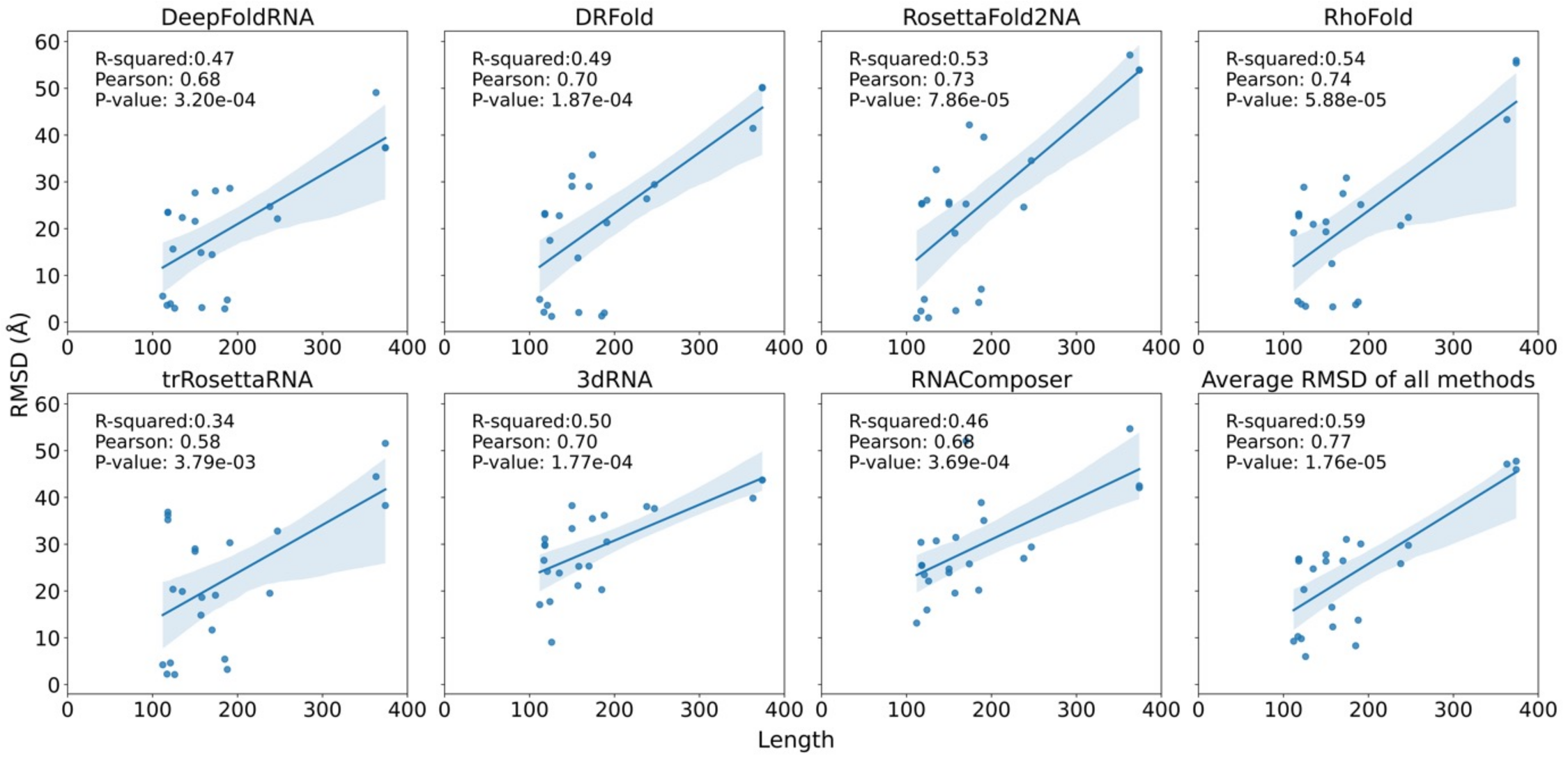

Supplement: S7 Fig — The correlation between the length of the target RNAs and the RMSD of the predicted model for all the methods. In this plot, only RNAs with length > 100 are considered. We see a positive correlation between RMSD and length indicating that as the RNA length increases the model quality decreases. (TIF) [file pcbi.1012715.s007.tif]

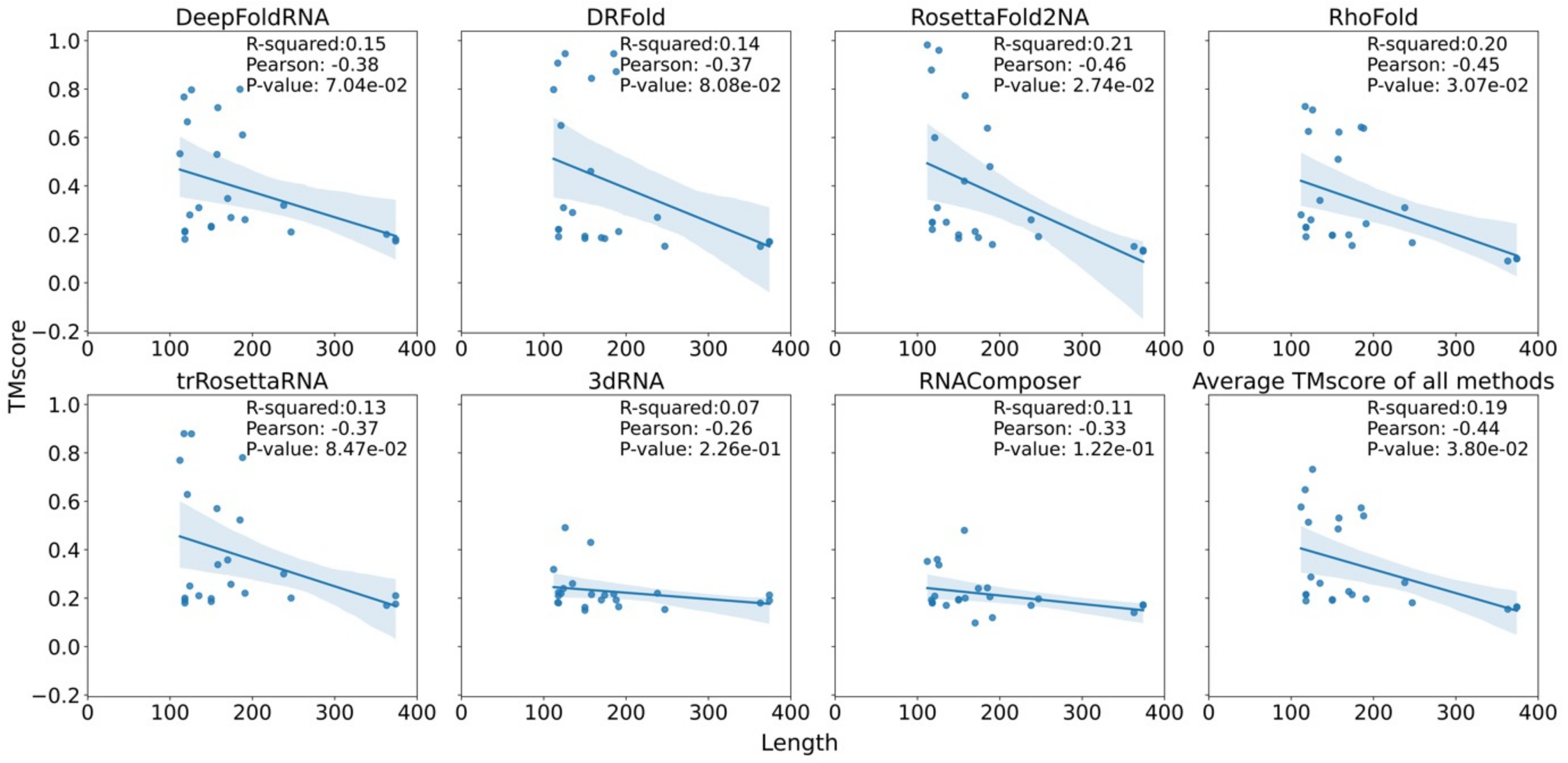

Supplement: S8 Fig — The correlation between the length of the target RNAs and the TMscore of the predicted model for all the methods. In this plot, only RNAs with length > 100 are considered. We see a negative correlation between RMSD and length indicating that as the RNA length increases the TMscore decreases, thus the model quality also decreases. (TIF) [file pcbi.1012715.s008.tif]

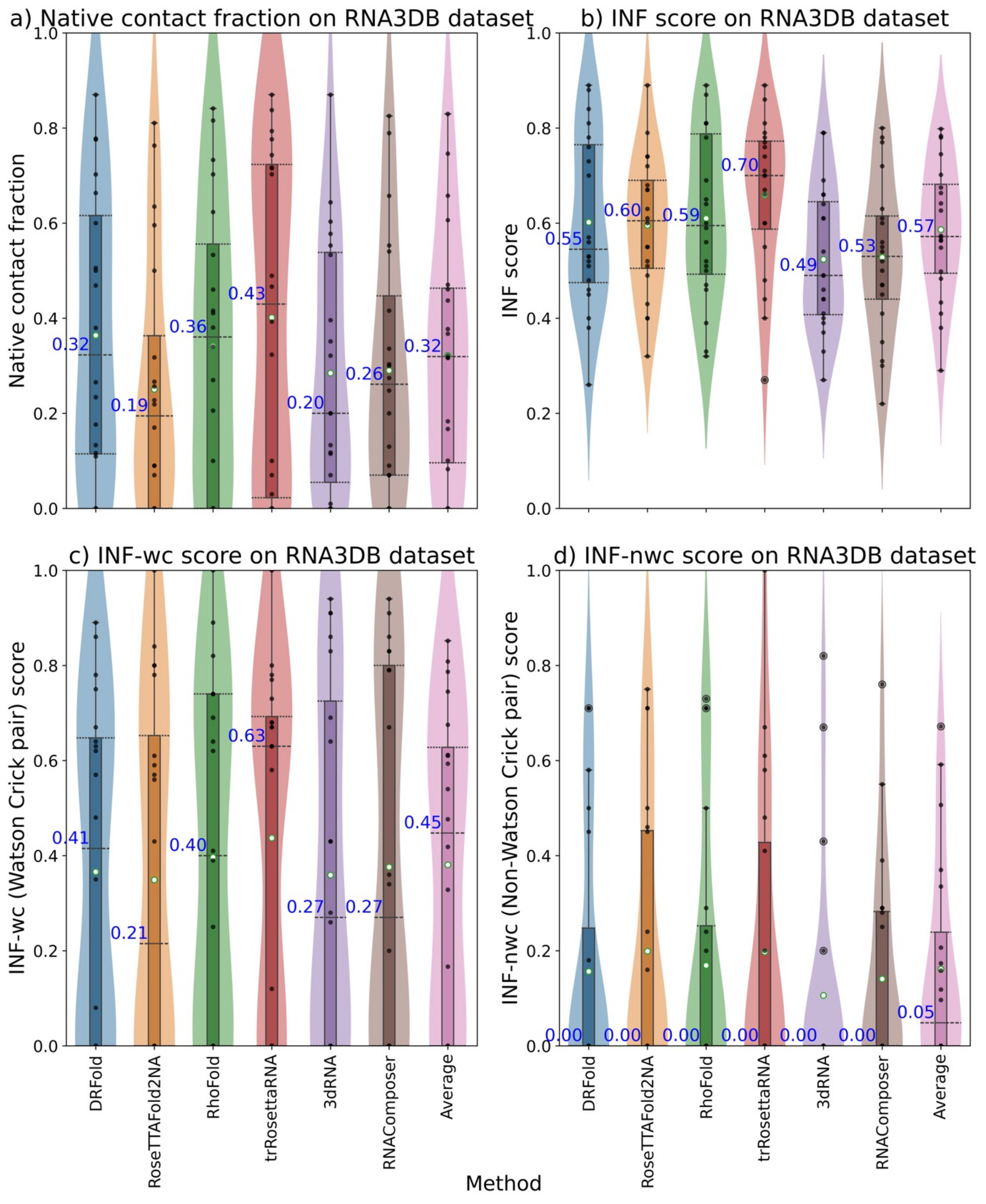

Supplement: S9 Fig — Results on the RNA3DB dataset. a) Native contact fraction (ncf) of the predicted models by the various methods. trRosettaRNA has the highest median ncf of 0.43. b) Interaction network fidelity (INF) score for the various methods. trRosettaRNA has the highest median INF score (0.70) c) INF-wc score (Watson-Crick pairs) for the various methods. trRosettaRNA has the highest median score of 0.63. Most of the methods have a low INF-WC score. d) INF-nwc score (non-Watson-Crick pairs) for the various methods. All of the methods have a median INF-nwc score of 0 (or close to 0), indicating that all of them fail to predict any non-canonical interaction pairs in these orphan RNAs. (TIF) [file pcbi.1012715.s009.tif]
